# Electronic Effects Drive Selectivity in CO_2_ Reduction Catalysis by Heptacoordinated Cobalt Complexes

**DOI:** 10.1021/acs.inorgchem.6c01633

**Published:** 2026-05-21

**Authors:** Federico Droghetti, Florian Lemken, Federico Castellani, Lubomír Rulíšek, Albert Ruggi, Mirco Natali

**Affiliations:** † Department of Chemical, Pharmaceutical and Agricultural Sciences, 9299 University of Ferrara, Via L. Borsari 46, Ferrara 44121, Italy; ‡ Institute of Organic Chemistry and Biochemistry of the Czech Academy of Sciences, Flemingovo Náměstí 2, Praha 6 160 00, Czech Republic; § Department of Chemistry, University of Fribourg, Chemin du Musée 9, Fribourg 1700, Switzerland

## Abstract

Controlling selectivity in CO_2_ reduction catalysis is essential for minimizing the formation of H_2_ byproduct. We recently reported that the cobalt and iron complexes of the 1-([2,2′-bipyridin]-6-yl)-*N*-([2,2′-bipyridin]-6-ylmethyl)-*N*-(pyridin-2-ylmethyl) methanamine ligand (**L**, DBPy-PyA) are efficient catalysts to selectively generate H_2_ and CO, respectively. Herein we demonstrate that selectivity can be controlled not only by exchanging the metal center but also adjusting the electronic properties of the ligand. Under electrochemical conditions in acetonitrile, with 2,2,2-trifluoroethanol as a proton source, the unsubstituted **CoL** and the electron-rich **CoL** ^**OMe**^ predominantly produce H_2_ (selectivity of 82% and 55%, respectively). In contrast, the electron-deficient **CoL** ^**CF3**^ favors CO formation with a selectivity up to 87%. DFT calculations show that formation of the metal-hydride is favored in the case of **CoL** and **CoL** ^**OMe**^, whereas it is substantially endergonic in the case of **CoL** ^**CF3**^. Concurrently, the binding of CO_2_ and the evolution of the resulting intermediates in **CoL** ^**CF3**^ can benefit from ligand-assisted proton transfer, as confirmed by microkinetic modeling. Catalytic tests were then conducted under photochemical conditions, affording syngas with tunable CO/H_2_ ratios that follow the electronic effects of the chosen catalyst. Overall, the results underscore how ligand tuning can be a powerful handle for controlling selectivity in molecular CO_2_RR.

## Graphical abstract

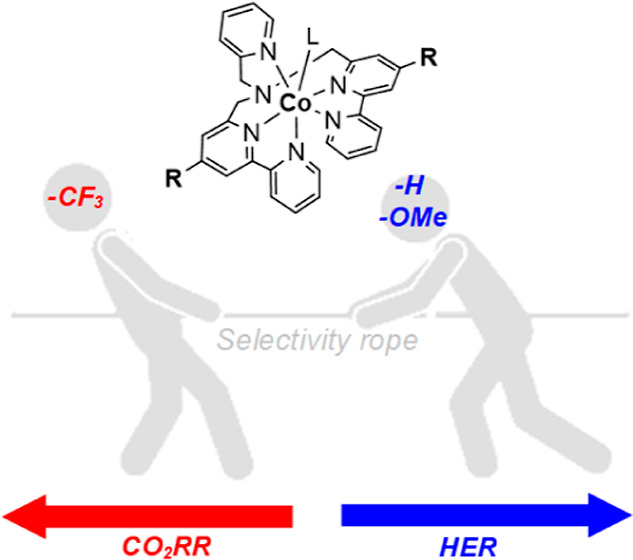

## Introduction

Global warming and climate change are among the most pressing environmental challenges of our time which originate from human activities such as burning fossil fuels, deforestation, and industrial processes.^1,2^ Central to these issues is the emission of CO_2_, a greenhouse gas that significantly contributes to the Earth’s rising temperatures.^3^ Converting CO_2_ into useful compounds, such as fuels or industrially relevant chemicals, can offer a promising solution for reducing CO_2_ levels and mitigating the impacts of climate change, paving the way for a sustainable future.^4–6^

Though simple at first glance, the CO_2_ reduction reaction (CO_2_RR) displays significant challenges. As a matter of fact, large energetic requirements need to be met for the one-electron reduction to its radical anion (eq 1),^7^ which could be in principle employed to fix CO_2_ into molecular scaffolds.^8^ More favorable reactivity can be attained in the presence of a proton donor,^9–11^ but at the expense of kinetic penalties associated with the multielectron and multiproton nature of the corresponding processes (eqs 2–6) as well as the possible competition among these latter reactions and the parallel hydrogen evolution reaction (HER, eq 7).

1 $${CO}_{2}+e^{-}\to {CO}_{2}^{\cdot -}$$

2 $${CO}_{2}+2H^{+}+2e^{-}\to CO+H_{2}O$$

3 $${CO}_{2}+2H^{+}+2e^{-}\to {HCO}_{2}H$$

4 $${CO}_{2}+4H^{+}+4e^{-}\to H_{2}CO+H_{2}O$$

5 $${CO}_{2}+6H^{+}+6e^{-}\to {CH}_{3}OH+H_{2}O$$

6 $${CO}_{2}+8H^{+}+8e^{-}\to {CH}_{4}+2H_{2}O$$

7 $$2H^{+}+2e^{-}\to H_{2}$$

Within this context, identifying active and selective catalysts poses as a fundamental objective for researchers working in the field of CO_2_RR.^12,13^ Molecular catalysts based on transition metal complexes have received considerable interest in the last years.^14–25^ Their catalytic activity mainly results in the generation of two-electron reduced products, namely CO or formate (eqs 2,3) and the parallel hydrogen byproduct (eq 7), although generation of higher reduced carbon-based species has been also documented.^26–28^

To control selectivity leading to a preferential generation of single products, various strategies have been considered with transition metal complexes as catalysts: exchange of the metal center,^24,29,30^ electronic or steric effects in the ligand scaffold,^31–34^ second-sphere effects,^35,36^ and change of the solvent.^37^ In this respect, the use of redox-active polydentate pyridine-based ligands^38^ has been exploited in several instances as a key strategy to shift the selectivity toward the formation of CO (eq 2). This is facilitated by the preferential delocalization of the reducing equivalents on the ligand framework which is expected to make the metal center less electron rich thereby favoring CO_2_ binding (leading to CO generation) over metal-hydride formation.^39^ The latter eventually leads to the production of formate or H_2_. Following these concepts, selective CO_2_ reduction into CO was achieved by Robert and co-workers using both the iron and cobalt complexes (Chart 1) of the tetradentate *qpy* ligand (where *qpy* = 2,2′:6′,2″:6″,2‴-quaterpyridine).^40^

**Chart 1:**
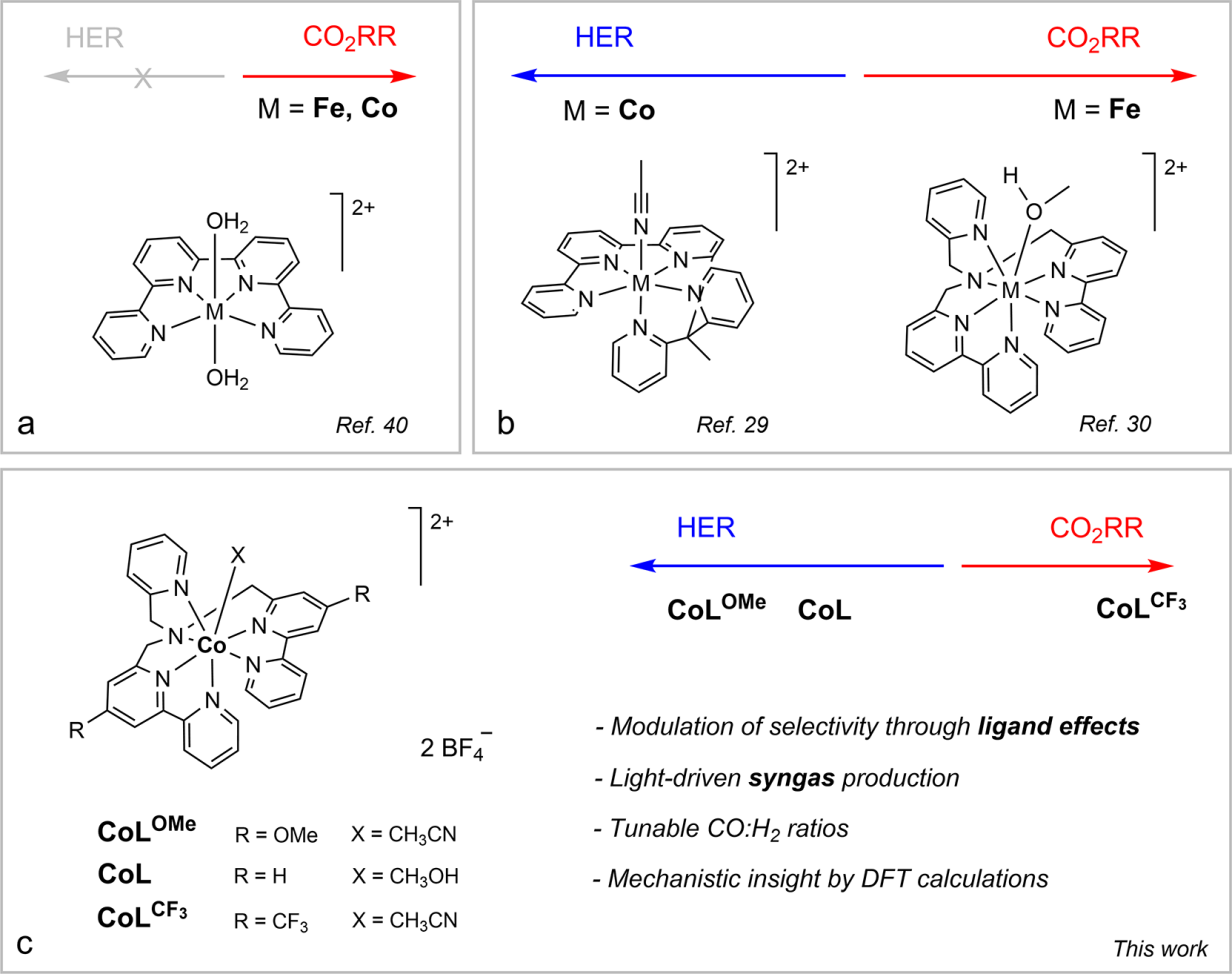
Molecular structures of (a) Co and Fe complexes showing selectivity for CO formation, (b) Co and Fe complexes showing selectivity for H_2_ and CO formation, respectively, and (c) Co complexes discussed in the present work where selectivity is modulated through electronic effects

While straightforward at first sight, this approach is not as general as expected, since the nature of the metal center has been shown to play a pivotal role in dictating catalytic selectivity. Chang and co-workers reported on electrochemical and light-driven catalytic studies involving the iron­(II) and cobalt­(II) complexes featuring the redox noninnocent pentadentate tpyPY2Me ligand (where tpyPY2Me = 6-(1,1-di­(pyridin-2-yl)­ethyl)-2,2′:6′,2″-terpyridine).^29,39,41^ While the iron complex in acetonitrile solution in the presence of 1 M phenol behaves as a selective catalyst toward CO_2_ conversion into CO, the cobalt analog delivers H_2_ as the major product,^29^ thus suggesting a critical role of the metal–ligand combination on selectivity. It is indeed very likely that the extent of conjugation and the actual involvement of the ligand in the reduction events might have an important influence on the reactivity of the reduced metal complex toward both the CO_2_ and proton substrates. Interestingly, we recently obtained consistent results when investigating the catalysis of the CO_2_RR in acetonitrile/water mixtures by the cobalt­(II) and iron­(II) complexes (Chart 1) of the redox-active, hexadentate DBPy-PyA ligand **L** (where DBPy-PyA = (1-([2,2′-bipyridin]-6-yl)-*N*-([2,2′-bipyridin]-6-ylmethyl)-*N*-(pyridin-2-ylmethyl)­methanamine). Both electrochemical and light-driven catalytic studies showed that CO is the major product for the iron complex, whereas H_2_ is mainly generated when employing the cobalt analog.^30^ Interestingly, further optimization of the reaction conditions using the iron complex as the catalyst allowed us to achieve 100% selective and efficient generation of CO in light-driven experiments.^42–44^

Prompted by these findings, we questioned whether the ability of our complexes based on the DBPy-PyA ligand to steer the reactivity toward CO or H_2_ is strictly dictated by the nature of the metal or can be modulated, using the same metal center, through electronic effects on the ligand scaffold. For these reasons, herein we investigate the activity toward the CO_2_RR in a protic environment of **CoL** and of two structural analogs, **CoL** ^**OMe**^ and **CoL** ^**CF3**^ (Chart 1), featuring electron-donating methoxy groups and electron-withdrawing trifluoromethyl groups in the bipyridine ligands, respectively. These complexes were previously investigated for the light-driven HER in aqueous media.^45–49^ Remarkably, the results show that modulation of the selectivity between CO formation and the parallel HER under electrochemical conditions can be attained also using cobalt as the metal center by fine-tuning the electronic structure of the ligand. Finally, when combined with a photosensitizer such as [Ru­(bpy)_3_]^2+^ (where bpy = 2,2′-bipyridine) or 1,3-dicyano-2,4,5,6-tetrakis­(diphenylamino)-benzene (4DPAIPN),^44^ photochemical production of syngas can be realized achieving different CO/H_2_ ratios of potential interest for diverse applications. By leveraging both experimental data and computational models, the present study will offer a robust and quantitative framework for understanding the underlying mechanisms that govern catalysis of the CO_2_RR by heptacoordinated polypyridine complexes.

## Results and Discussion

### Electrochemical Properties

The cyclic voltammograms (CVs) of the **CoL**, **CoL** ^**CF3**^, and **CoL** ^**OMe**^ complexes in acetonitrile solution under Ar display two different reversible reduction features (Figure 1a and Table S1). The first one, occurring at half-wave potentials of *E*_1/2_ = –1.50, −1.29, and −1.59 V vs Fc^+^/Fc for **CoL**, **CoL** ^**CF3**^, and **CoL** ^**OMe**^, respectively, is a one-electron process. The second, featured at more negative potentials, is instead associated with a two-electron process. In this regard, while a single wave is recorded in the case of **CoL** and **CoL** ^**OMe**^ (half-wave potentials of *E*_1/2_ = −2.06 and −2.13 V vs Fc^+^/Fc, respectively), two consecutive waves are observed in the case of **CoL** ^**CF3**^ featuring half-wave potentials of *E*_1/2_ = −1.73 and −1.85 V vs Fc^+^/Fc. Importantly, the potential values of both processes are affected by the chemical nature of the substituents.^47^ In the case of the trifluoromethyl electron-withdrawing group (EWG), a significant positive shift can be recorded in comparison with the unsubstituted **CoL**. On the other hand, when the methoxy electron-donating group (EDG) is present, a pronounced cathodic shift is experienced for both reduction processes.

**Figure 1:**
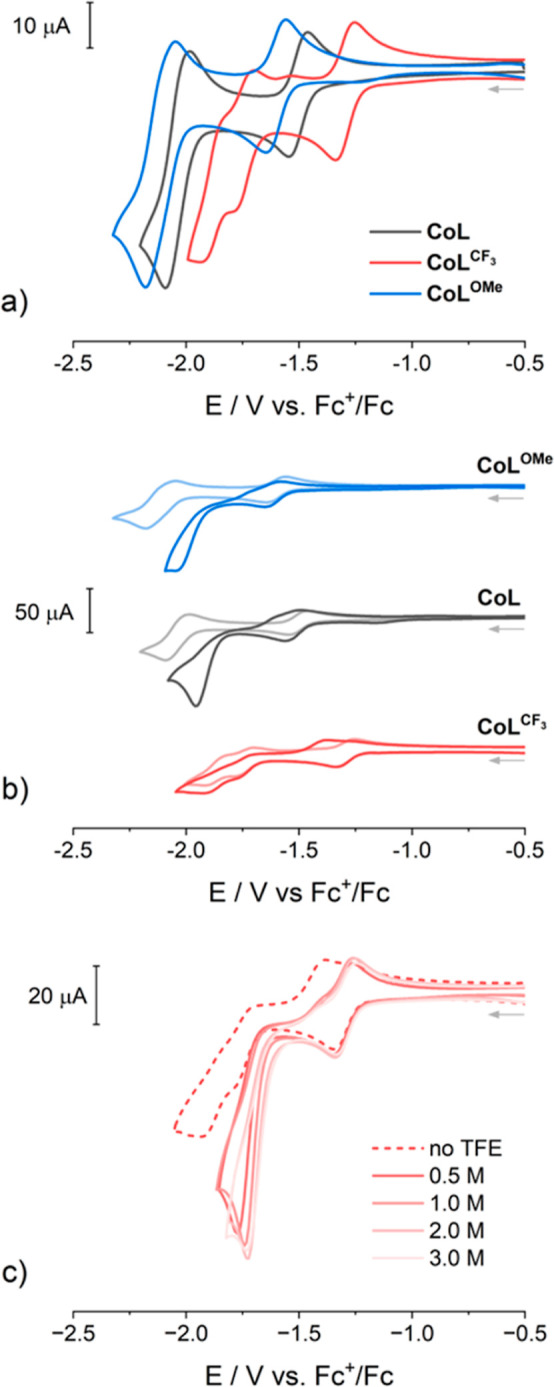
(a) CVs of 1 mM complexes **CoL**, **CoL** ^**CF3**^ and **CoL** ^**OMe**^ (0.1 M TBAPF_6_) under Ar; (b) comparison of the CVs for the three complexes in acetonitrile under Ar (light trace) or under CO_2_ (dark trace); (c) CVs of 1 mM complex **CoL** ^**CF3**^ in acetonitrile under CO_2_ in the presence of 0–3 M TFE (all CVs were measured at room temperature and at a scan rate of 0.1 V/s).

DFT calculations of the reduction potentials of the one- and two-electron reduced species are in excellent agreement (mean absolute error of ca. 40 mV, see Table S2) with the experimental *E*_1/2_ values of both reduction waves in the case of **CoL** and **CoL** ^**OMe**^ and of the first two waves in the case of **CoL** ^**CF3**^, lending credence to the employed computational protocol for relatively complex electrochemical steps that follow the initial reduction in the overall process. Løwdin population analysis (Figures S7 and S8) is qualitatively identical for all three investigated complexes and indicate that the reduction steps occur predominantly on the DBPy-PyA ligand^30,42^ whereas the cobalt ions remain formally in a +2 oxidation state, although Løwdin populations indicate that the effective partial charge of the cobalt ions is close to +1. In particular, within the quartet ground state of the two-electron reduced systems, the spins of the unpaired electrons on the DBPy-PyA ligand are oriented in an antiparallel fashion with respect to each other, formally leaving three unpaired electrons of mutually parallel spin at the metal center. On the other hand, in the near-degenerate doublet state (w.r.t. the quartet state) the spins of the ligand-centered electrons are oriented parallel to each other but antiparallel to the three unpaired electrons located on the metal center. The sextet state, in which all five unpaired electrons have parallel spins, was found to be considerably higher in energy for all investigated complexes. For the sake of simplicity, we will use cobalt formal oxidation states as indicators of the level of reduction of the complex, understanding from the above that the reduction might not occur on the metal center.

To get a deeper insight into the structural properties of the metal complexes and of the resulting one- and two-electron reduced species in acetonitrile solution, the Gibbs free energy associated with the binding of a solvent molecule to the metal center was computed (an acetonitrile molecule was considered for all complexes). The formation of the metal–ligand bond is already slightly endergonic before reduction of the complexes (computed Δ*G* = +1.3, +0.4, and +0.9 kcal·mol^–1^ for **CoL**, **CoL** ^**CF3**^, and **CoL** ^**OMe**^, respectively) and becomes significantly endergonic after the first reduction (Δ*G* = +9.0, +11.1, and +8.4 kcal·mol^–1^, for **CoL**, **CoL** ^**CF3**^, and **CoL** ^**OMe**^, respectively). Furthermore, spontaneous breaking of the metal–ligand bond after the second reduction prevents calculation of the exact free energy change under the employed computational methodology. Thus, it can be concluded that, upon dissolution, only in the starting form of the cobalt complexes is the acetonitrile solvent molecule, if at all, weakly bonded to the cobalt center. Its detachment is indeed a prerequisite to open up the coordination site to enable substrate activation.

### Electrochemical CO_2_ Reduction

Having obtained the initial electrochemical and computational characterization of the redox properties of the studied complexes, we further assessed the CV response in CO_2_-saturated acetonitrile solution (Figure 1b). For the **CoL** and **CoL** ^**OMe**^ complexes, a slight current increase (*i*_cat_/*i*_p_ ∼1.2) is experienced at the first reduction wave, suggesting a sluggish reactivity of the one-electron reduced species (associated with a formal Co­(I) intermediate hereafter) with the CO_2_ substrate.^30^ On the other hand, a detectable current enhancement is apparent for all complexes near the second reduction event. Interestingly, in the case of **CoL** and **CoL** ^**OMe**^ the catalytic half-wave potential (*E*_cat_, calculated at the inflection point of the wave)^50^ displays an anodic shift with respect to the *E*_1/2_ of the second reduction event in the absence of the CO_2_ substrate (Table S1), indicating a fast chemical reaction^51^ between the two-electron reduced species (associated with a formal Co(0) intermediate hereafter) and CO_2_. For both these complexes, the observed current increase (*i*_cat_/*i*_p_ = 4.9 and 5.1 for complexes **CoL** and **CoL** ^**OMe**^, respectively) at the potential of the Co­(I)/Co(0) process suggests the ability to promote CO_2_ reduction to CO through an EECC mechanism (E indicates an electron-transfer and C a chemical reaction), where CO_2_ here acts as both the substrate and oxide acceptor.^52^ In this regard, the larger potential shift observed for **CoL** ^**OMe**^ over **CoL** (150 and 130 mV, respectively) also suggests that the second chemical step is faster in the former,^51^ in agreement with the presence of EDGs. This is further corroborated by the observation of an anodic process at −1.7 V vs Fc^+^/Fc during the return scan, likely associated with the oxidation of catalytic intermediates,^30,52^ only in the case of complex **CoL**. In the case of **CoL** ^**CF3**^ no current increase is observed at the first cathodic process, whereas weak current enhancements are detected at the second and third cathodic waves (*i*_cat_/*i*_p_ = 1.7 and 2.5, respectively). For these processes, the catalytic half-wave potentials (*E*_cat_) fall at identical values of the corresponding *E*_1/2_ of the triggering redox couples (Table S1), pointing to a slow reactivity^51^ with CO_2_ of the reduced cobalt complex featuring trifluoromethyl EWGs.

The CVs of the complexes were further examined in a CO_2_ atmosphere in the presence of 2,2,2-trifluoroethanol (TFE) as a proton donor (p*K*_a_ = 34.5 in acetonitrile).^53^ For all the complexes investigated, the addition of increasing amounts of TFE (Figure 1c for **CoL** ^**CF3**^, Figures S1 and S2 for **CoL** and **CoL** ^**OMe**^, respectively) leads to an additional current enhancement. At 3 M TFE, an *i*_cat_/*i*_p_ of 11.3, 4.5, and 10.1 can be estimated for **CoL**, **CoL** ^**CF3**^, and **CoL** ^**OMe**^, respectively, confirming that the presence of the TFE proton donor accelerates catalytic CO_2_ reduction. The less intense current enhancement experienced by **CoL** ^**CF3**^ is consistent with a weaker reactivity toward CO_2_, as expected based upon the presence of the EWGs as previously inferred. Nevertheless, for all complexes a parallel shift of the catalytic wave toward more positive potentials is observed in the presence of TFE highlighting how the proton donor also enables a substantially faster reaction between the two-electron reduced species and the CO_2_ substrate. Accordingly, CO_2_ reduction in the presence of TFE is expected to occur through an EECC mechanism for all the complexes examined, leading to the formation of CO according to the reaction mechanism in Scheme 1a. However, given the concurrent ability of all the complexes to form a metal-hydride species upon reduction,^45–49^ it can be envisaged that the current increase can also be potentially induced by parallel catalytic reactions such as the formation of formate or the hydrogen byproduct (Scheme 1b).

**Scheme 1:**
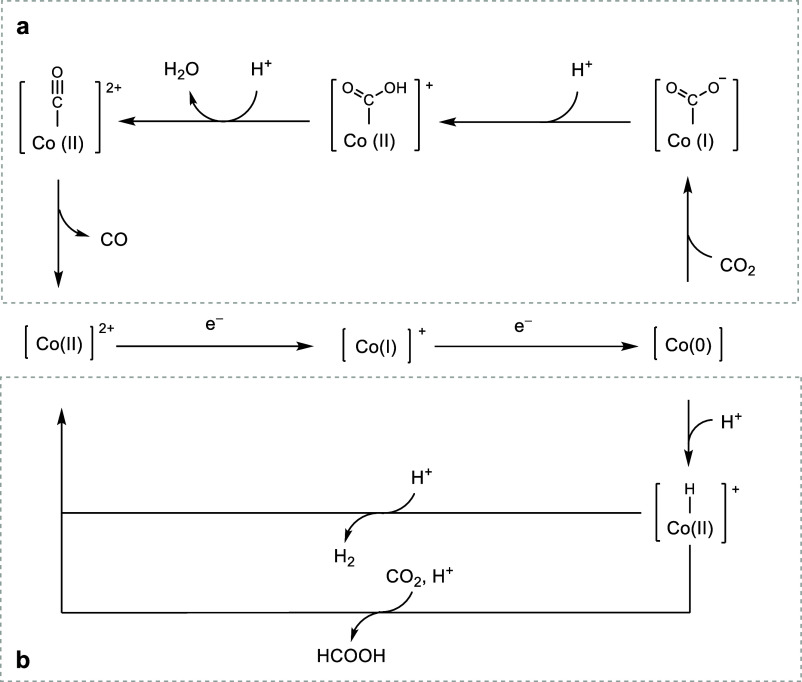
Proposed Mechanism for Catalytic (a) CO and (b) Formate or H_2_ Formation by Complexes **CoL**, **CoL** ^**CF3**^ and **CoL** ^**OMe**^ in Acetonitrile Solution in the Presence of a Proton Donor

Controlled potential electrolysis (CPE) experiments were performed to identify and quantify the reduction products. Gaseous products such as CO and H_2_ were detected by gas chromatography, while liquid products were identified by NMR. As to the latter, only formate was found. CPE results for all complexes are collected in Table 1, while the product spectrum is represented in a schematic form in Figure 2. Inspection of the data shows that **CoL** delivers H_2_ as the major product (Entry 1), with a selectivity of 82%, while the CO_2_ reduction products (CO and HCOOH) are obtained with low FE. These results do not differ substantially from those observed using **CoL** in the presence of 10% H_2_O as the proton donor,^30^ indicating the strong tendency of the complex toward the hydride pathway (Scheme 1b). H_2_ remains the primary product for the **CoL** ^**OMe**^ complex (selectivity of 55%), with only a minor amount of CO produced akin to the unsubstituted analog (selectivity of 19%). These results suggest that the introduction of EDGs in the DBPy-PyA ligand has minor effects on catalyst selectivity (Entry 2). Indeed, although the electron-rich metal center might exhibit enhanced reactivity with the CO_2_ substrate, as previously indicated by CV data without TFE, the formation of the metal-hydride is also anticipated to be highly favorable in the presence of the proton donor. In this regard, the larger amount of formate produced by **CoL** ^**OMe**^ with respect to **CoL** (selectivity of 36%) suggests a more favorable reactivity of the corresponding metal-hydride with CO_2_.

**Table 1:** Summary of the CPE Results^a^

entry				FE/%			selectivity/%^c^		
	**CAT**	**E/V** ^b^	**[TFE]/M**	**CO**	**H**_**2**_	**formate**	**CO**	**H**_**2**_	**formate**
1	**CoL**	–2.0	1	9.5	57.7	3.4	13	82	5
2	**CoL** ^**OMe**^	–2.1	1	5.9	16.8	7.9	19	55	26
3	**CoL** ^**CF3**^	–1.9	0.5	19.8	1.5	1.6	87	6	7
4	**CoL** ^**CF3**^	–1.9	1	26.1	4.6	3.6	76	14	10
5	**CoL** ^**CF3**^	–1.9	2	21.3	4.6	3.5	73	16	11

a Data are estimated after 2 h, 1 mM catalyst concentration, CO_2_-saturated acetonitrile solution (0.1 M TBAPF_6_), glassy carbon rod as working electrode, Pt as counter electrode, SCE as reference.

b Potentials are converted vs Fc^+^/Fc by subtracting 0.4 V to the applied potential vs SCE.^54^

c Estimated as the ratio between the amount of each product and the total amount of products.

**Figure 2:**
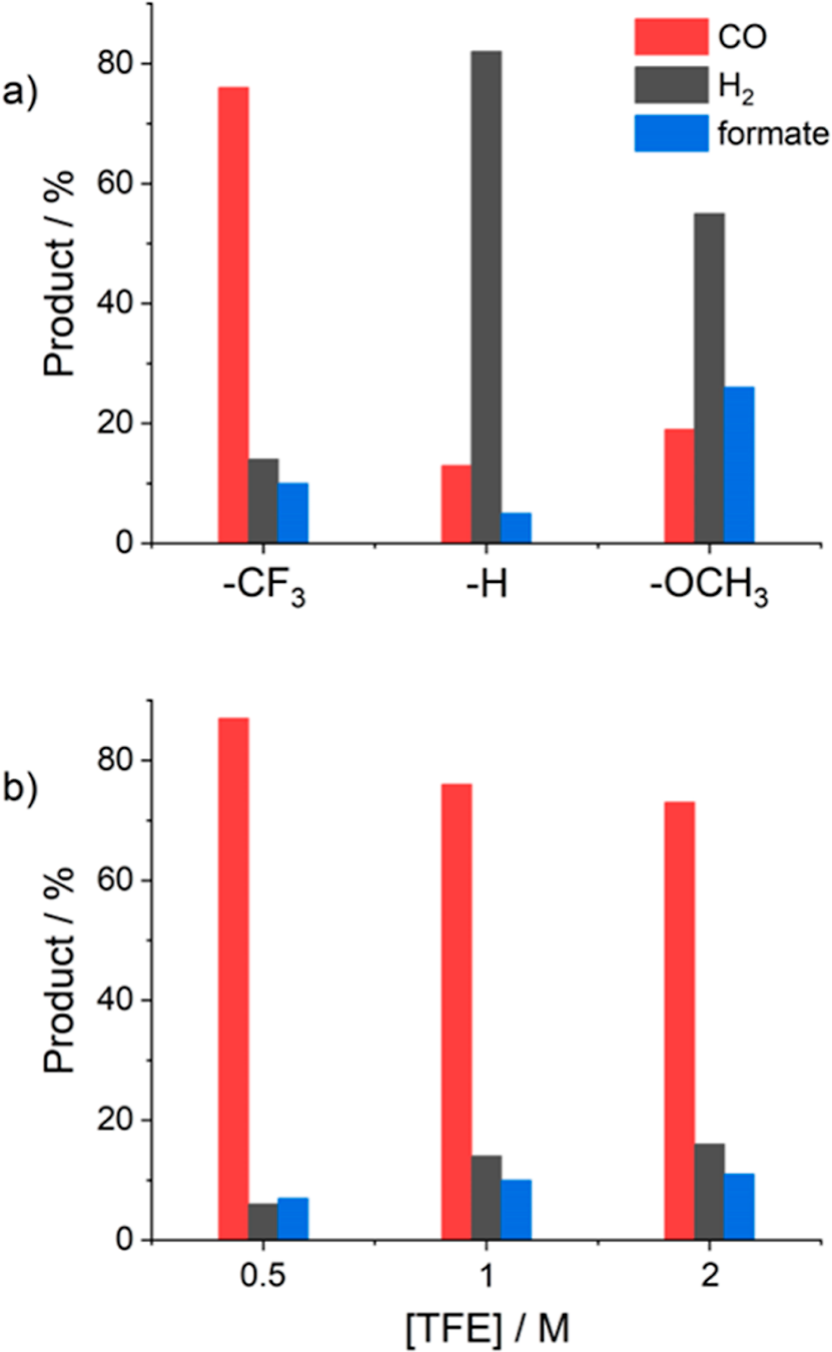
Selectivity of product formation obtained after 2 h CPE of (a) 1 mM complexes **CoL**, **CoL** ^**CF3**^ and **CoL** ^**OMe**^ in acetonitrile (0.1 M TBAPF_6_) under CO_2_ in the presence of 1 M TFE and (b) 1 mM **CoL** ^**CF3**^ in the presence of variable concentrations of TFE.

A different behavior can be observed in the case of **CoL** ^**CF3**^ for which CO instead represents the major product (selectivity of 76% at 1 M TFE, Entry 4). This can be rationalized considering that, although the introduction of EWGs on the DBPy-PyA ligand might slow down the reactivity with CO_2_, it is likely that the reaction with the proton donor becomes even slower. This, in turn, favors the carboxyl pathway (Scheme 1a) over the competing hydride formation (Scheme 1b) thereby leading to selective CO production. We next evaluated the effect of the proton donor concentration on the catalytic activity of **CoL** ^**CF3**^ (Entries 3–5). Formation of CO becomes even more selective (87%) upon lowering the TFE concentration, whereas higher amounts of both H_2_ and formate result at higher TFE loadings (Figure 2b). Both these findings can be explained considering that the formation of the cobalt-hydride intermediate becomes more favorable when the concentration of the proton donor increases.

For all complexes, control tests conducted after CPE confirm that the observed catalytic activity does not originate from heterogeneous species formed on the electrode surface.^55,56^ As a matter of fact, CV analysis of an unpolished GC electrode in a catalyst-free solution confirms the absence of any current enhancement potentially associated with catalytic activity (Figures S3–S5). Consistently, when a previously used GC electrode (from CPE experiments with all complexes) was subjected to bulk electrolysis at the same potential in a catalyst-free solution, only residual currents and trace amounts of H_2_ were detected.

In summary, these results prove that the modulation of the electronic density on the DBPy-PyA ligand using cobalt as the metal center can impact catalyst selectivity. Specifically, while EDGs retain the inherent propensity of the unsubstituted complex toward H_2_ production, EWGs instead promote high selectivity for CO generation. This tunability very likely comes from an asymmetric influence that the chemical substituents on the bipyridine ligands exert on the reactivity of the reduced cobalt center toward either CO_2_ or protons. This shift is very likely attributable to a more effective suppression of proton vs CO_2_ binding at the two-electron reduced cobalt intermediate, eventually favoring CO_2_ activation and CO formation.

### Computational Studies

The catalytic mechanism for both CO and H_2_/formate formation were studied using DFT-D3//COSMO-RS calculations (see Materials and methods below and Section S1 of the Supporting Information for methodological details). In both cases, we explored the EECC pathways, which are anticipated to be the predominant ones according to the electrochemical experiments reported above. The mechanistic proposal for the CO_2_RR to CO, which aligns with the mechanisms suggested in various studies on similar catalytic systems,^33,39,41,42,57^ along with the computed thermodynamic and kinetic parameters, is schematically illustrated in Scheme 2. The parallel HER mechanism (including also formate generation) is instead depicted in Scheme 3. Figure 4 finally summarizes the energy landscape associated with both the CO_2_RR to CO and the competitive HER under the explored experimental conditions.

**Scheme 2:**
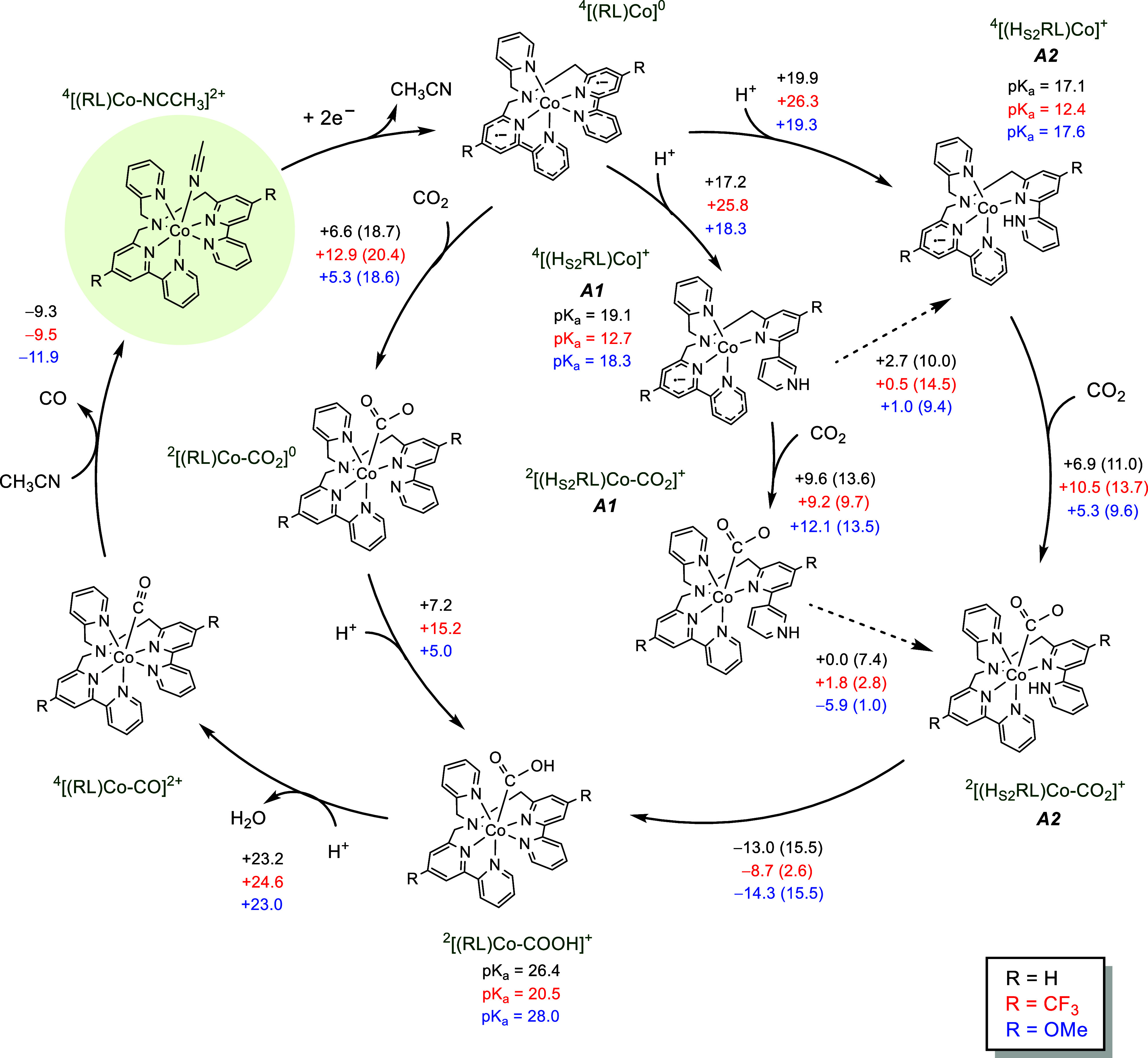
Proposed Reaction Mechanism of the Catalytic Cycle of the CO_2_RR for Our Cobalt Catalysts Calculated at the B3LYP-D3­(BJ)/COSMO-RS/def2-TZVPD Level of Theory (the Starting Point is Highlighted in Green, for the Sake of Clarity)^a^–^c^

a Reaction free energies (Δ*G*), in kcal·mol^–1^, of individual reaction steps are printed along the reaction arrows for R = H (black), for R = CF_3_ (red), and for R = OMe (blue).
b Free energies of associated transition states (Δ*G* ^‡^), in kcal·mol^–1^, are added in parentheses.
c p*K*_a_ values of protonated intermediates are referred to acetonitrile. Free energies of intermolecular PTs assume the deprotonation of TFE.

**Scheme 3:**
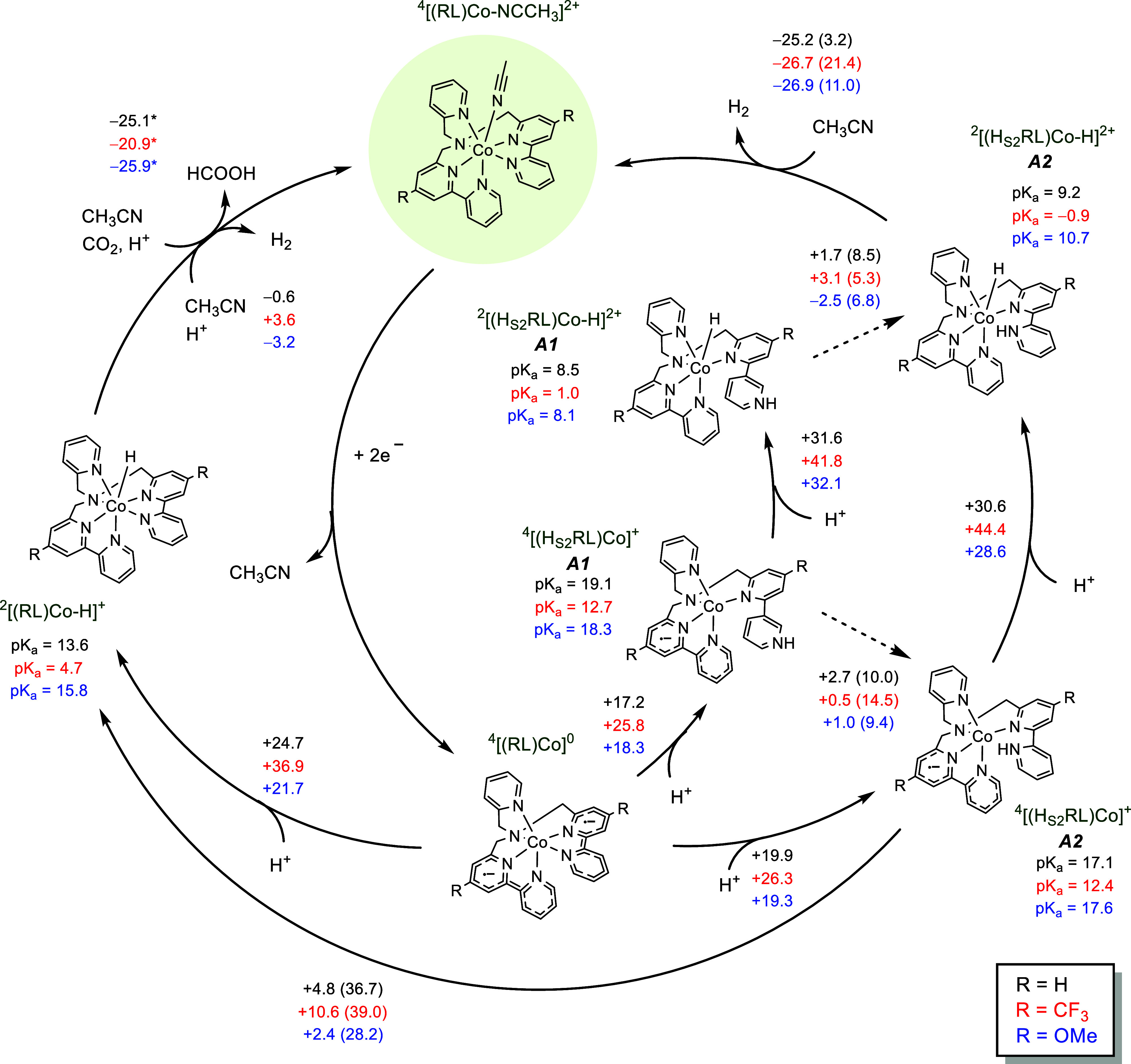
Proposed Reaction Mechanism of the Catalytic Cycle of the HER Reaction and Formate Formation for Our Cobalt Catalysts Calculated at the B3LYP-D3­(BJ)/COSMO-RS/def2-TZVPD Level of Theory (the Starting Point is Highlighted in Green, for the Sake of Clarity)^a^–^d^

a Reaction free energies (Δ*G*) of individual reaction steps, in kcal·mol^–1^, are printed along the reaction arrows for R = H (black), for R = CF_3_ (red), and for R = OMe (blue).
b Free energies of associated transition states (Δ*G* ^‡^), in kcal·mol^–1^, are in parentheses.
c p*K*_a_ values of protonated intermediates are referred to acetonitrile. Secondary pathways involving ligand protonation are displayed on the right. Free energies of intermolecular PTs assume the deprotonation of TFE.
d Free energies with the asterisk (*) refer to the formation of formate, not formic acid.

**Figure 3:**
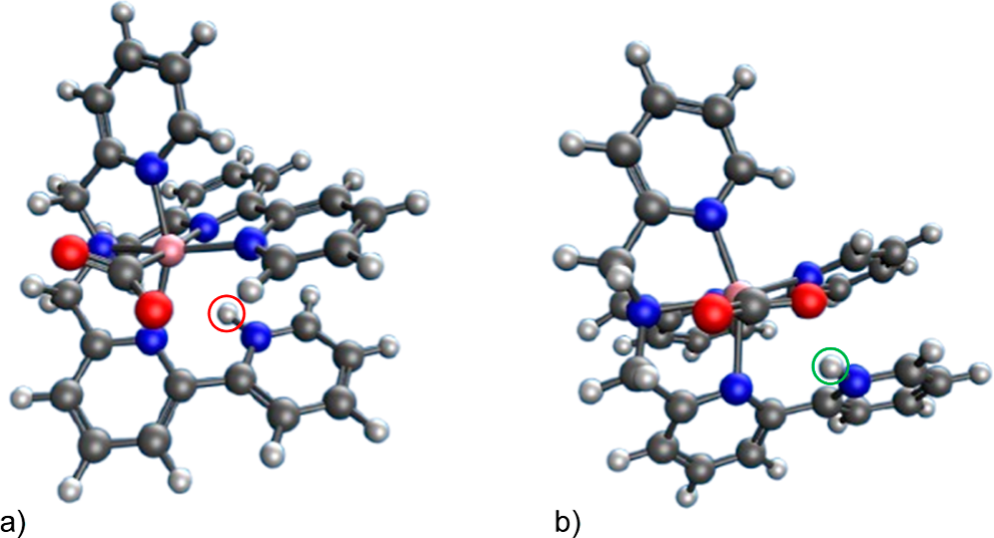
Front view on the coordination site in conformer (a) **A1** and (b) **A2** of ligand protonated **CoL** featuring a bonded CO_2_ (the relevant proton is highlighted with a circle for clarity).

**Figure 4:**
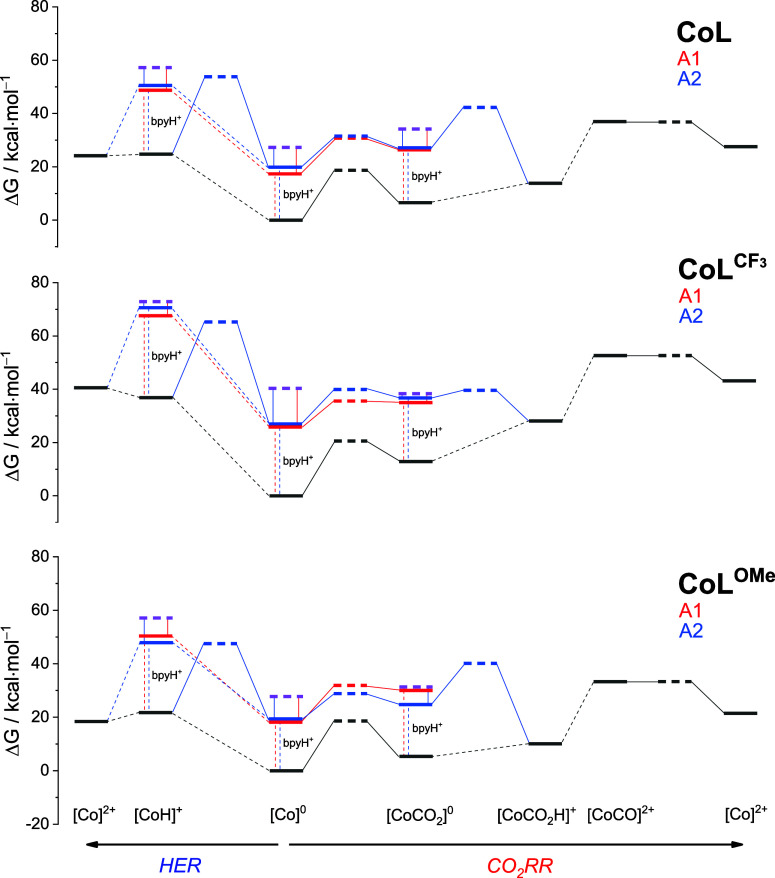
Gibbs free energy profiles of the CO_2_RR to CO and HER without ligand protonation (black) and with protonation of the *bpy* (blue or red, depending on the conformer). Formate formation is omitted due to the small fraction attained in the product spectrum. Free energies of intermolecular PTs to the catalyst are given in reference to TFE. Intermediates are marked by solid lines, transition states are denoted by dashed lines (purple lines are transition states for **A1**/**A2** interconversion), dashed connecting lines indicate that a transition state cannot be determined under the employed methodology.

### CO_2_ Binding

The first step of the proposed EECC mechanism is the binding of CO_2_ to the two-electron reduced catalyst [(RL)­Co]^0^ resulting in the hexacoordinated species [(RL)­Co–CO_2_]^0^. Computations indeed indicate that a distal pyridine of one *bpy* which faces the coordination site is only weakly bonded to the cobalt center (Figure S9). Addition of CO_2_ stabilizes the detachment of this moiety, which in turn presumably stabilizes the CO_2_ adduct. Similar cases of hemilabile pyridine groups in the context of the CO_2_RR have been discussed in the literature.^17^ Despite this stabilization, the binding of CO_2_ is endergonic for all three investigated catalysts. The computed binding free energies for **CoL** and **CoL** ^**OMe**^ were found to be very similar at Δ*G* = +6.6 and +5.3 kcal·mol^–1^. In contrast, binding of CO_2_ to the two-electron reduced **CoL** ^**CF3**^ complex appears considerably more endergonic (Δ*G* = +12.9 kcal·mol^–1^), consistent with a lower Lewis basicity due to the presence of EWGs. Computed kinetic barriers (activation free energies) for the addition of CO_2_ were, however, found to differ negligibly among the three complexes, with **CoL** ^**OMe**^ featuring the lowest barrier at Δ*G* ^‡^ *=* 18.6 kcal·mol^–1^, and **CoL** ^**CF3**^ the highest at 20.4 kcal·mol^–1^. These data indicate that the presence of EWGs and EDGs in the DBPy-PyA ligand has only a minor effect on the transition state of the CO_2_ addition. Thus, this step is expected to equilibrate with approximately similar rates for all complexes, but the equilibrium will be considerably more on the side of unbound CO_2_ for **CoL** ^**CF3**^ than for **CoL** and **CoL** ^**OMe**^, consistent with the lower current enhancement observed in the CV of **CoL** ^**CF3**^ (*vide supra*).

### Protonation of Bonded CO_2_

The second step of the catalytic mechanism is the protonation of the bonded CO_2_ molecule. Calculated p*K*_a_ values of the resulting metal carboxylic acids (i.e., [(RL)­Co–COOH]^+^ in Scheme 2) are consistent with expectations based on Lewis basicity. They indicate that **CoL** ^**OMe**^ is most prone to protonation of the bonded CO_2_, followed closely by **CoL**, while **CoL** ^**CF3**^ is the least favorable. It is worth noting that the calculated p*K*_a_ value of 28.0 computed for **CoL** ^**OMe**^ is similar to that of the employed proton donor (calculated p*K*_a_ = 31.7 for TFE in acetonitrile), suggesting an efficient proton transfer (PT).

### Dehydroxylation

Protonation of the metal carboxylic acid [(RL)­Co–COOH]^+^ results in the detachment of water forming the metal carbonyl [(RL)­Co–CO]^2+^ shown in Scheme 2. No energy minimum corresponding to the structure with the protonated metal carboxylic acid intermediate could be found, as water is consistently removed during geometry optimizations for all complexes. This indicates barrier-free detachment of water occurring concertedly with the PT, i.e., a one-step dehydroxylation process.

### CO Release

CO is released from the metal carbonyl [(RL)­Co–CO]^2+^, recovering the original species [(RL)­Co–NCCH_3_]^2+^. While calculations at a lower level of theory (BP86-D3/def2-TZVP) predicted marginal reaction barriers for this process, higher level calculations (B3LYP-D3­(BJ)/def2-TZVPD) consistently indicate barrier-free detachment of CO for all complexes (see Figure S10 for the potential energy surface of the Co–CO bond stretching). Due to the lack of reaction barriers, we conclude that the detachment of CO occurs concertedly with the preceding dehydroxylation step. Therefore, the catalysts do not suffer from the slow product release that was found to severely impede related catalytic systems.^40,58–60^ In addition, fast and efficient CO release prevents the formation of more stable metal carbonyls by additional electrochemical reduction, which could inhibit product release and thus inactivate the catalyst.^52^

### HER Mechanism

We further theoretically investigated the EECC mechanism leading to the formation of H_2_ and formate (Scheme 3). The essential reaction step in this mechanism is the formation of a metal hydride [(RL)­Co–H]^+^ via PT to the metal center. Either product (formate or H_2_) is then expected to be formed by insertion of CO_2_ or H^+^ into the metal-hydride bond, respectively. The p*K*_a_ value calculated for the **CoL** hydride (13.6) is slightly lower than for **CoL** ^**OMe**^ (15.8), while the value for **CoL** ^**CF3**^ is considerably lower (4.7). This indicates that the propensity to form the hydride species is similar for both **CoL** and **CoL** ^**OMe**^, whereas it is considerably attenuated for **CoL** ^**CF3**^, as can be inferred from its lower Lewis basicity due to the presence of EWGs. In particular, the calculated p*K*_a_ value of the hydride of **CoL** ^**CF3**^ is so low compared to the employed proton donor (Δp*K*_a_ = 27.0 corresponding to a Δ*G* = +36.8 kcal·mol^–1^) that its formation can be anticipated to be severely hindered under the employed experimental conditions. Hence, we conclude that, if on one hand the presence of EWGs in **CoL** ^**CF3**^ lowers its activity toward the CO_2_RR (see above), on the other hand it definitely suppresses the reactivity toward the HER to a much greater extent. Moreover, the final step of the HER is exergonic for **CoL** and **CoL** ^**OMe**^, but endergonic for **CoL** ^**CF3**^. Both these findings thus provide a valuable explanation for the high selectivity of **CoL** ^**CF3**^ toward the reduction of CO_2_ to CO, as experimentally observed.

### Protonation of DBPy-PyA Ligand

Importantly, the aforementioned hemilability of the DBPy-PyA ligand (*vide* CO_2_ binding) leaves a detached pyridine prone to protonation.^61^ This enables the DBPy-PyA ligand to act as a proton shuttle.^13,42,57,62^ This situation can significantly alter the mechanistic pathways described above by changing the environment around the metal center and providing alternative, intramolecular pathways for the protonation of key catalytic intermediates. Although computations indicate that protonation of the DBPy-PyA ligand is consistently endergonic with respect to deprotonation of TFE, calculated p*K*_a_ values for the protonated ligand (Schemes 2 and 3) were found to be also consistently higher than those of their corresponding metal hydrides, indicating that protonation of the ligand is indeed more likely than protonation of the metal center. Interestingly, the protonated pyridine is very flexible, and our computational study identified two distinct low-energy conformers (Figures 3 and S9), hereafter referred to as **A1** and **A2**, characterized by very similar acidity but vastly different reactivity. In **A1** the distal ring of the protonated *bpy* moiety is positioned underneath the second *bpy* moiety, stabilized by dispersion interactions between the aromatic rings. Thus, the added proton faces away from the coordination site and cannot interact with any species bonded there. On the other hand, in **A2** the added proton is oriented toward the coordination site, allowing for H-bonding and PT to the metal center or a bonded substrate molecule. Remarkably, the conversion between the two conformers is predicted to be considerably slower than PT from the **A2** conformer to a bonded CO_2_ molecule (Scheme 2 for the associated reaction barriers).

It should be noted that either protonated species features a considerably broadened coordination site compared to the deprotonated catalysts at the cost of a reduced Lewis basicity resulting from increased overall charge. Consequently, the sterically unhindered formation of the metal hydrides becomes significantly less efficient after ligand protonation. This can be quantified by the increased acidity of the metal hydrides [(H_S2_RL)­Co–H]^2+^ with a protonated ligand compared to [(RL)­Co–H]^+^ without a protonated ligand (Scheme 3). Among the different catalysts and conformers, PT to the ligand indeed increases the acidity of the metal hydrides by values ranging from −3.7 to −7.7 p*K*_a_ units. In contrast, the binding of CO_2_ after ligand protonation becomes only mildly more endergonic for **CoL** (ΔΔ*G* = +0.3 and +3.3 kcal·mol^–1^ for **A1** and **A2**, respectively) and **CoL** ^**OMe**^ (ΔΔ*G* = +7.2 and +0.0 kcal·mol^–1^), and even less endergonic for **CoL** ^**CF3**^ (ΔΔ*G* = −3.7 and −2.4 kcal·mol^–1^), whereas kinetic barriers for CO_2_ addition decline substantially for all catalysts (Scheme 2). The attenuation of the activation barrier for CO_2_ addition is most pronounced for the **A1** conformer of **CoL** ^**CF3**^, dropping from +20.4 kcal·mol^–1^ without ligand protonation to only +9.7 kcal·mol^–1^. Regarding intramolecular PTs from the protonated DBPy-PyA ligand (**A2** conformer) to the metal center or CO_2_ bonded thereat, computations indicate prohibitively high kinetic barriers for the former (Δ*G* ^‡^ = 28.2–39.0 kcal·mol^–1^, Scheme 3) but comparatively shallow barriers for the latter PT (Δ*G* ^‡^ = 2.6–15.5 kcal·mol^–1^, Scheme 2). Remarkably, PT from the ligand to the carboxylate group is particularly efficient for the **CoL** ^**CF3**^ complex (Δ*G* ^‡^ = 2.6 kcal·mol^–1^), indicating rapid formation of the metal carboxylic acid after addition of CO_2_ to ligand-protonated **CoL** ^**CF3**^. Hence, we expect that ligand protonation plays an important role in the high selectivity of **CoL** ^**CF3**^ toward CO_2_ reduction to CO. In contrast, it is anticipated to have a strong retarding effect on the parallel HER.

### Kinetic Modeling

To attain further insight into the reaction mechanisms and the effects promoted by the substituents on the catalytic activity of our cobalt complexes, we performed microkinetic modeling of the catalytic cycles represented in Schemes 2 and 3. Herein, the energy of the transition states of intermolecular PTs were approximated via a Marcus relation,^63,64^ using a range of intrinsic reaction barriers; the formation of formate was also neglected due to its low fraction in the experimental product spectrum. Within this framework, rate-determining steps were identified using Campbell’s degree of rate control^65–67^ (vide Section S1 of the Supporting Information for methodological details). For the HER mechanism, formation of the metal hydride is indicated to be the rate limiting step, particularly for high intrinsic reaction barriers, i.e., for slow intermolecular PT rates (Figures S11 and S12). For the CO_2_RR to CO, our microkinetic model shows that CO_2_ binding exhibits a negligible degree of rate control for all catalysts, indicating rapid equilibration of this reaction step (Figure S13), which is consistent with previous studies.^41,53^ The two following intermolecular PTs, on the other hand, feature very similar, high degrees of rate control regardless of the intrinsic reaction barrier (Figures S14 and S15). These data are consistent with the detection of reaction intermediates of the CO_2_RR in the return scan of the CV (see Figure 1). Most importantly, our kinetic model predicts that ligand protonation has a key effect on the catalytic activity of **CoL** ^**CF3**^ (Figure S16). In particular, at intermediate intrinsic reaction barriers, ligand protonation shows a strong decelerating effect (negative degree of rate control) on the HER, while concomitantly exhibiting a significant positive degree of rate control over the CO_2_RR. Thus, our kinetic model further supports the beneficial effect of ligand protonation within the catalytic mechanism of **CoL** ^**CF3**^ in favoring the CO_2_RR to CO over the parallel HER, highlighting once again the critical role of the ligand in assisting PT events via intramolecular routes.

### Light-Driven CO_2_ Reduction

The activity of the cobalt complexes **CoL**, **CoL** ^**CF3**^, and **CoL** ^**OMe**^ toward the CO_2_RR was further investigated under light-driven conditions. These experiments were conducted using a three-component photochemical system combining the metal complexes with a light-harvesting sensitizer and an electron donor.^13,62^ *N*,*N*-diisopropylethylamine (DIPEA) was chosen as the sacrificial electron donor, whereas two different sensitizers were tested, namely [Ru­(bpy)_3_]^2+^ and 4DPAIPN.^43,44,68,69^ The latter was indeed shown to promote more efficient and selective CO formation over the commonly used ruthenium sensitizer when combined with the parent iron complex.^43^

For both systems, activation of the catalyst is expected to occur following photoexcitation (eq 8) and involves reductive quenching of the excited sensitizer (PS) by the DIPEA (eq 9) and subsequent electron transfer from the photogenerated PS^–^ species to the catalyst, either in its initial (eq 10) or one-electron reduced species (eq 11). Chemical steps finally occur (eqs 12–14 depending on the resulting products) according to the reaction mechanisms depicted in Schemes 2 and 3.

8 $$PS+h\nu \to {PS}^{*}$$

9 $${PS}^{*}+DIPEA\to {PS}^{-}+{DIPEA}^{\cdot +}$$

10 $${PS}^{-}+Co\left(II\right)\to PS+Co\left(I\right)$$

11 $${PS}^{-}+Co\left(I\right)\to PS+Co\left(0\right)$$

12 $$Co\left(0\right)+{CO}_{2}+2H^{+}\to Co\left(II\right)+CO+H_{2}O$$

13 $$Co\left(0\right)+{CO}_{2}+2H^{+}\to Co\left(II\right)+HCOOH$$

14 $$Co\left(0\right)+2H^{+}\to Co\left(II\right)+H_{2}$$

The photochemical experiments were carried out under continuous irradiation with a 460 nm LED of a CO_2_-saturated acetonitrile solution containing 0.4 mM PS, 0.1 M DIPEA, and 50 μM of cobalt complex in the presence of 1 M TFE. A lower catalyst concentration of 10 μM was also considered in the case of the Ru-based sensitizer.^30^ Control experiments confirm that all components are required and that CO_2_ is the source of reduced carbon products (Table S4). Under the conditions tested, H_2_ and CO are the main products. A small quantity of formate is indeed only found using the [Ru­(bpy)_3_]^2+^ sensitizer, which is comparable to that attained in blank experiments in the absence of the catalyst (Table S4), ascribable to catalysis promoted by catalytically active species originating from the sensitizer after removal of a *bpy* ligand, as already reported.^70,71^ Thus, we refrain from considering formate as a possible product of light-driven catalysis by the cobalt complexes examined, and the following discussion will be limited, for sake of simplicity, to the sole gaseous products. The kinetics of product formation are reported in the Supporting Information (Section S4, Figures S17–S22). All the results in terms of amount of H_2_ and CO formed, the corresponding turnover number (TON), turnover frequency (TOF), and quantum yield (Φ) are collected in Table 2. A schematic comparison in terms of maximum TONs is also provided in Figure 5 for 50 μM catalyst concentration.

**Table 2:** Summary of the Light-Driven Catalytic Results^a^

entry				n/μmol (TON)^b^		TOF/h^–1^ (QY/%)^c^		selectivity/%^d^	
	**Cat**	[Cat]/μM	**PS**	**CO**	**H**_**2**_	**CO**	**H**_**2**_	**CO**	**H**_**2**_
1	**CoL**	50	[Ru(bpy)_3_]^2+^	10.2 (41)	47.8 (191)	37 (2.7)	154 (11.3)	17	83
2	**CoL** ^**CF3**^	50	[Ru(bpy)_3_]^2+^	12.7 (51)	31.2 (125)	41 (3.0)	95 (7.0)	29	71
3	**CoL** ^**OMe**^	50	[Ru(bpy)_3_]^2+^	6.7 (27)	61 (244)	28 (2.1)	175 (12.8)	10	90
4	**CoL**	10	[Ru(bpy)_3_]^2+^	6.7 (134)	18.9 (378)	138 (2.0)	336 (4.9)	26	74
5	**CoL** ^**CF3**^	10	[Ru(bpy)_3_]^2+^	4.3 (86)	7.1 (142)	156 (2.3)	312 (4.6)	38	62
6	**CoL** ^**OMe**^	10	[Ru(bpy)_3_]^2+^	6 (120)	53 (1060)	198 (2.9)	1164 (17.1)	10	90
7	**CoL**	50	4DPAIPN	12 (48)	42 (168)	21 (1.5)	60 (4.4)	22	78
8	**CoL** ^**CF3**^	50	4DPAIPN	23.5 (94)	28.8 (115)	29 (2.1)	50 (3.7)	45	55
9	**CoL** ^**OMe**^	50	4DPAIPN	16.7 (67)	63 (252)	26 (1.9)	94 (6.9)	21	79

a 460 nm LED of CO_2_-purged acetonitrile solutions containing 0.4 mM PS, 0.1 M DIPEA, 1 M TFE, (the data reported are averages of two independent experiments).

b estimated at the plateau of the kinetic trace (see Supporting Information).

c estimated in the linear portion of the kinetic trace.

d estimated as the ratio between the amount of a single product and the total amount of products both taken at the plateau.

**Figure 5:**
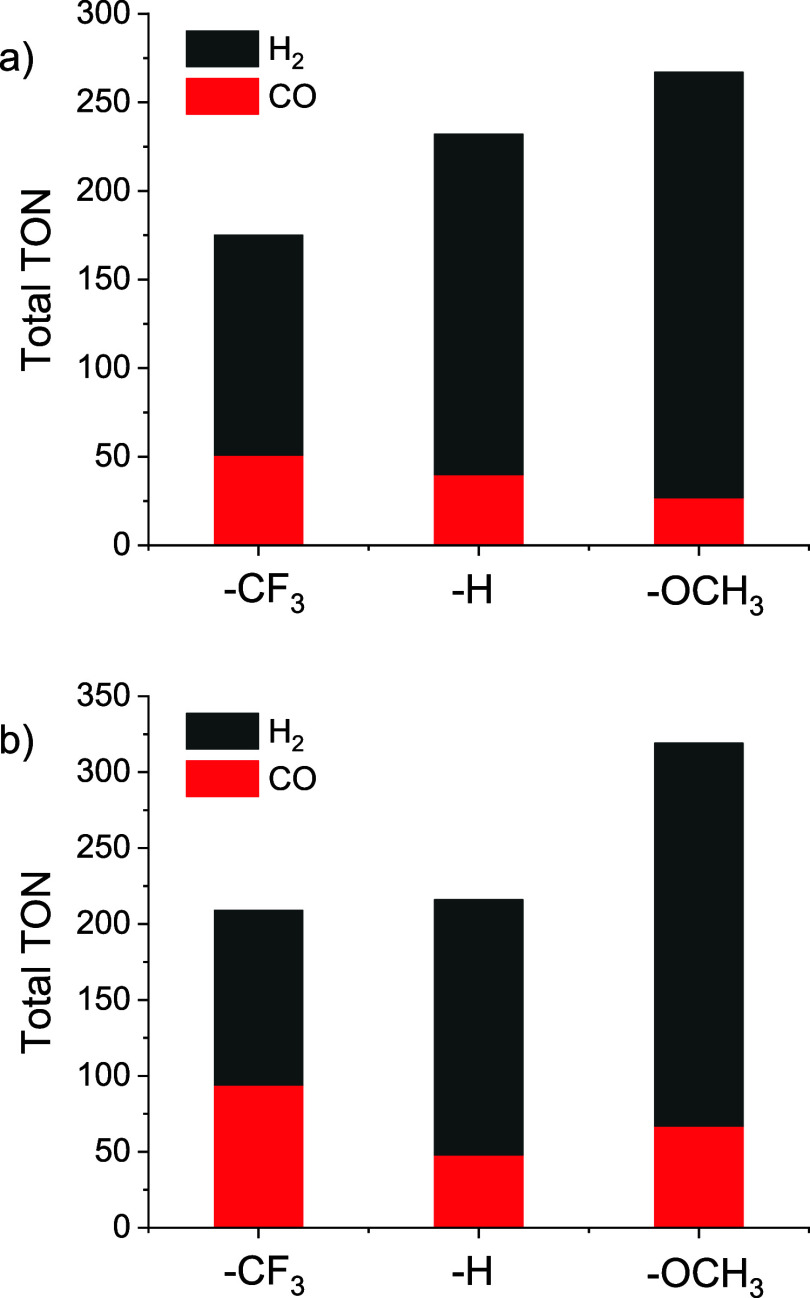
Total TON of H_2_ and CO formation obtained upon 460 nm LED irradiation of acetonitrile solutions containing 50 μM complexes **CoL**, **CoL** ^**CF3**^ and **CoL** ^**OMe**^, 0.1 M DIPEA, and 1 M TFE under CO_2_ in combination with (a) 0.4 mM [Ru­(bpy)_3_]­(PF_6_)_2_ and (b) 0.4 mM 4DPAIPN sensitizers.

Using the [Ru­(bpy)_3_]^2+^ sensitizer, all the complexes yield H_2_ as the major product with maximum TONs of 191, 125, and 244 and quantum yields (Φ) of 11.3%, 7.0%, and 12.8% for **CoL**, **CoL** ^**CF3**^, and **CoL** ^**OMe**^, respectively, at 50 μM catalyst concentration (Entries 1–3 in Table 2). Under these conditions, CO is only obtained as a minor product with maximum TONs of 41, 51, and 27 and quantum yields (Φ) of 2.7%, 3.0%, and 2.1%, respectively. The selectivity toward H_2_ formation progressively decreases from 90% to 71% in the order **CoL** ^**OMe**^ **> CoL** > **CoL** ^**CF3**^. At a lower catalyst concentration of 10 μM (Entries 4–6), a similar product distribution is observed, with H_2_ remaining the dominant product. Notably, a slight increase in both TON and TOF for both products is generally achieved despite lower absolute amounts reached at the plateau.

Interestingly, qualitatively similar results were obtained also using the organic sensitizer 4DPAIPN (Entries 7–9 in Table 2) for which a slight gain in terms of overall CO produced is mainly recorded with a selectivity increasing from 21% to 45% in the order **CoL** ^**OMe**^ **< CoL** < **CoL** ^**CF3**^.

The absence of quantitative CO formation and the negligible production of formate observed for **CoL** ^**CF3**^ reflect distinct catalytic scenarios accessed under photochemical vs electrochemical conditions, in line with recent literature.^71,72^ As a matter of fact, under light-driven catalysis, the photooxidation of the DIPEA electron donor (eqs 8,9) is followed by deprotonation leading to the formation of diisopropylethylammonium.^73^ Notably, this species is a substantially stronger acid than TFE (p*K*_a_ ∼19 in acetonitrile compared to 35.8 for TFE).^53,74^ Under these circumstances, metal hydride formation in **CoL** ^**CF3**^ becomes more feasible by a factor of ∼22 kcal·mol^–1^ and can approach the free energy of the CO_2_ coordination step (see Schemes 2 and 3 for the relevant thermodynamic quantities), thereby biasing the reaction landscape toward the hydride pathway. Consequently, H_2_ evolution can compete with CO formation, thus explaining the generation of a syngas mixture. Likewise, the reaction of the hydride intermediate with CO_2_, which governs the selectivity between H_2_ and formate, is strongly influenced by the p*K*_a_ of the acid source, with stronger acids favoring H_2_ formation over formate.^30,42,75^ Overall, the photochemical results underscore the sensitivity of product selectivity to proton source and reaction protocol.^76^

Importantly, a closer inspection of the results in Table 2 and Figure 5 clearly reveals that substituent electronic effects unequivocally play a decisive role in governing the light-driven catalytic selectivity. Specifically, the CO/H_2_ ratio systematically increases in the order **CoL** ^**OMe**^ **< CoL** < **CoL** ^**CF3**^, closely mirroring the progressively electron-withdrawing nature of the substituents on the DBPy-PyA ligand framework. It is also worth highlighting that the increase of the CO yield is obtained at the expense of a decrease in the overall formation of reduced products (H_2_ + CO), more relevant at lower catalyst concentrations, consistent with a progressive decrease in reactivity of the reduced metal center with lowering electron donation from the ligand. Thus, the observed trend well complies with theoretical predictions still highlighting the critical role of ligand modification on catalytic reactivity and selectivity.

Remarkably, exploiting the cobalt complexes here described within light-driven reaction schemes offers a means to the production of syngas with tunable CO/H_2_ ratios ranging from 1:9 (obtained using [Ru­(bpy)_3_]^2+^ and **CoL** ^**OMe**^) up to an almost 1:1 ratio (employing 4DPAIPN and **CoL** ^**CF3**^). This flexibility is particularly advantageous for tailoring syngas composition to suit specific downstream applications. For instance, a CO-rich syngas is preferred in Fischer–Tropsch synthesis and methanol production,^77^ whereas a H_2_-rich mixture is more suited for combustion purposes.^78^ The ability to tune the CO/H_2_ ratio by photochemical means through the modulation of the sensitizer/catalyst combination represents an interesting tool in the pursuit of sustainable fuel production.

## Materials and Methods

### Experimental Section

All reagents were obtained from standard suppliers and used without additional purification. The cobalt complexes **CoL**, **CoL** ^**OMe**^, and **CoL** ^**CF3**^ were prepared following our established synthetic protocol and their chemical characterization perfectly match the data reported in the original publications.^45,47^ [Ru­(bpy)_3_]­(PF_6_)_2_ was obtained by salt metathesis from the [Ru­(bpy)_3_]­Cl_2_·6H_2_O, while 4DPAIPN was available from a previous study.^43^ *No uncommon hazards are noted.* Details of the instrumental apparatuses and procedures used for the electrochemical and photochemical studies are provided in the Supporting Information (see Section S1).

### Computational Section

Geometry optimizations and electronic structure calculations were performed using the Turbomole-7.7 program package.^79^ Geometry optimizations were performed at the BP86-D3­(BJ)/def2-TZVP level of theory,^80–88^ and electronic structure calculations at the B3LYP-D3­(BJ)/def2-TZVPD (or ωB97M-V/def2-TZVPD if explicitly stated) level of theory.^80–92^ Vibrational frequencies were calculated with Turbomole-7.7 and converted into vibration free energies with the thermo submodule of the XTB software package.^95^ Solvation free energies were computed using the COSMO-RS method,^93,94^ that combines the conductor-like solvation model (COSMO) and statistical fluid thermodynamics, using the COSMOtherm23 software package.^95^ Spin multiplicities of the ground electronic states were determined from the dependency of the spin-crossover energies on the exact exchange fraction.^96^ For this linear regression we used the BP86, PBE,^88,97^ B3LYP*,^98–100^ B3LYP and PBE0 functionals,^88,97,101^ and the exact spin multiplicity of the ground state was determined at an exact exchange fraction of 15%, corresponding to the specialized B3LYP* ^102–104^ or PBE0* ^105,106^ functionals, which were found to reproduce high-quality CASPT2 + δMRCI spin-crossover energies for similar metal complexes with high accuracy.^105,106^ Following the reaction-coordinate-driven transition-state search method,^107,108^ one-dimensional PESs were calculated analogous to geometry optimizations. Microkinetic modeling was performed with the Tenua 2.1 software package.^109^ Further details on the computational methodologies are provided in Section S1 of the Supporting Information.

## Conclusions

In this work, we have investigated the catalytic ability toward CO_2_RR of a series of heptacoordinated cobalt complexes differing in the presence of electron-withdrawing or electron-donating groups. Under electrochemical conditions in acetonitrile solution in the presence of TFE as a proton donor, the electron-rich complexes **CoL** and **CoL** ^**OMe**^ favor the formation of H_2_ whereas the electron-poor analog **CoL** ^**CF3**^ delivers CO as the major product, showing how the electronic effect of the substituent is a key factor governing selectivity. In this regard, the switch from H_2_ to CO formation using **CoL** ^**CF3**^ can be achieved thanks to an asymmetric effect imparted by the electron-withdrawing substituents which more strongly disfavor proton binding at the reduced metal center over CO_2_ binding. Quantum chemical calculations provided good agreement with experimental data and consistently reproduce qualitative experimental trends, emphasizing the importance of hemilability of the DBPy-PyA ligand for CO_2_ binding. In this regard, microkinetic analysis indicates that, for the **CoL** ^**CF3**^ catalyst, ligand protonation exerts opposite effects on the HER and CO_2_RR pathways by decelerating metal hydride formation, while enhancing PT to the metal carboxylate intermediate thereby accelerating CO formation. The catalytic activity was further assayed under light-driven conditions using [Ru­(bpy)_3_]^2+^ and 4DPAIPN as the light-harvesting sensitizers. Under these conditions, photochemical generation of syngas with different CO/H_2_ ratios was attained, featuring progressively larger values in the order **CoL** ^**OMe**^ < **CoL** < **CoL** ^**CF3**^, i.e., following the tendency previously predicted by both electrochemical and computational studies. The improved CO selectivity observed for **CoL** ^**CF3**^ is, however, accompanied by a modest decrease in the total TON of reduced products, underscoring the intimate connection between catalytic activity and selectivity. All in all, the possibility to tune the catalytic activity in heptacoordinated metal complexes by both metal swapping^30,42^ and/or electronic effects definitely highlights the great potential of this family of molecular catalysts as promising components in solar fuel generation.

## Supplementary Material





## Notes

Additional electrochemical, computational, and photochemical data (PDF)
Computational data: coordinates of all stationary points and their respective energies (ZIP)

## ⊥

Institute of Inorganic Chemistry of the Slovak Academy of Sciences, Dúbravská cesta 9, 845 36, Karlová Ves, Slovak Republic
