# Supplementary material for: Electronic Effects Drive Selectivity in CO2 Reduction Catalysis by Heptacoordinated Cobalt Complexes
Source: Inorg Chem. 2026 May 21;65(22):12678–92. doi: 10.1021/acs.inorgchem.6c01633 (PMC13250995; doi:10.1021/acs.inorgchem.6c01633)
Supplement: Supplementary file 1 [file ic6c01633_si_001.pdf]

Supporting Information

Electronic Effects Drive Selectivity in CO_2 Reduction Catalysis by Heptacoordinated Cobalt Complexes

Federico Droghetti,[‡,∥] *Florian Lemken,*[†,∥,♦] *Federico Castellani,*[‡] *Lubomír Rulíšek,*[†]*
Albert Ruggi,[⁋]* *and Mirco Natali*[‡]*

[‡] Department of Chemical, Pharmaceutical and Agricultural Sciences, University of Ferrara, Via L. Borsari 46, 44121 Ferrara, Italy. E-mail: mirco.natali@unife.it

[†] Institute of Organic Chemistry and Biochemistry of the Czech Academy of Sciences, Flemingovo náměstí 2, 160 00, Praha 6, Czech Republic. E-mail: lubomir.rulisek@uochb.cas.cz

[⁋] Department of Chemistry, University of Fribourg, Chemin du Musée 9, 1700 Fribourg, Switzerland. E-mail: albert.ruggi@unifr.ch,

[∥] These authors contributed equally.

[♦] Current address: Institute of Inorganic Chemistry of the Slovak Academy of Sciences, Dúbravská cesta 9, 845 36, Karlová Ves, Slovak Republic.

Table of content

S1. Methodology p. S2

S1.1. Experimental section p. S2

S1.2. Computational methodology p. S3

S2. Electrochemical section p. S6

S3. Computational section p. S9

S4. Photochemical section p. S23

S5. References of the Supporting Information p. S29

S1. Materials and methods

S1.1 Experimental section

Absorption spectra were recorded at room temperature using an Agilent Technologies spectrophotometer. Cyclic voltammetry (CV) measurements were performed on a PGSTAT302N potentiostat (Autolab) in a three-electrode cell, using a glassy carbon as the working electrode, a silver wire as the quasi-reference electrode, and a Pt wire as counter electrode. $TBAPF_6$ was employed as the supporting electrolyte. Solutions were purged for 20 min with either N_2 or CO_2 before analysis. Controlled potential electrolysis (CPE) experiments were performed in a gas-tight custom-made electrochemical cell using a high-surface area glassy-carbon rod as the working electrode, a platinum wire as the counter electrode (separated from the test solution by a frit) and SCE as the reference electrode (potentials were subsequently converted vs. Fc^+/Fc for uniformity with CV experiments by subtracting 0.4 V).[S1] The head-space of the cell was connected to a gas-chromatography (GC) apparatus (see below for details) for the determination and quantification of H_2 and CO. The bulk electrolysis experiments were run in duplicate, and the results reported are averages of two independent experiments. Light-driven CO_2 reduction reaction (CO_2RR) experiments were performed under continuous irradiation using a 3 W Blue LED at 460 nm.[S1] In a typical photochemical experiment, samples were prepared in 20 mL scintillation vials by mixing stock solutions of the sensitizer and the catalysts, followed by the addition of DIPEA and proton source. The solution was then placed in the reactor, purged with CO_2 for 20 minutes, and continuously stirred. Experiments were run in duplicate, and the results are reported as the average of two independent experiments. The measuring cell (both in cases of the bulk electrolysis and or photochemical cell) is sealed during the reaction: the head to which the cell is attached has four ports, closed with Swagelok® connections, two of them are part of a closed loop involving GC gas inlet and sample vent to analyze the headspace content without an appreciable gas consumption, and the other two are for the degassing procedure (input and output). The gas phase of the reaction vessel was analyzed on an AgilentTechnologies490 micro-GC equipped with a 5 Å molecular sieve column (10 m), a thermal conductivity detector, and using Ar as carrier gas. The unused gas sample is then reintroduced in the reactor to minimize its consumption along the whole photolysis. The moles of gases were quantified through the external calibration method. In the case of H_2, this procedure was performed through a galvanostatic (typically 1 mA) electrolysis of a 0.1 M H_2SO_4

solution. A 100% faradaic efficiency (FE) was assumed leading to a linear correlation between the amount of H_2 evolved at the cathode and the electrolysis time. CO was quantified using a response factor obtained by injecting known amounts of gas in the cell and then sampling the headspace. Average errors are within ±10% for CO and ±5% for H_2. Formate was quantified using ^{1}H-NMR (δ=8.3 ppm). The electrolyzed or photolyzed solutions were brought to basic pH upon the addition of the minimum amount of NaOH and then evaporated under reduced pressure. The solid residue was then dissolved in 2 mL D_2O and sonicated for 5 min, dimethylformamide (1 µL, δ=7.8 ppm) was then added as an internal standard and the resulting solution was filtered before entering the NMR tube.

S1.2 Computational methodology

All Gibbs free energies G calculated in this work include: electronic energy, vibrational free energy, and solvation free energy (eq. S1). The Gibbs free energies corresponding to a particular structure/molecule in the solvent (S) can be then conveniently expressed as:

$$G_S = E_{el} + \Delta G_{solv} + E_{ZPVE} - RT\ln(q_{trans}q_{rot}q_{vib}) + pV \qquad \text{(S1)}$$

where E_{el} is the electrostatic potential energy of the molecule in vacuo (gas-phase molecular energy), ΔG_{solv} is the solvation energy, E_{ZPVE} is the zero-point vibrational energy whereas $RT\ln(q_{trans}q_{rot}q_{vib})$ are the entropic terms obtained from the rigid-rotor/harmonic oscillator (RRHO) approximation in which a free rotor model was applied for low-lying vibrational modes under 100 cm^{-1} with a smoothing function applied (sometimes denoted as quasi-RRHO, or RRFRHO approximation). The standard states used are 1 atm, T = 298.15 K and 1 $mol\cdot L^{-1}$. In particular, geometry optimizations and electronic structure calculations were performed using the Turbomole-7.7 program package.[S3] Geometry optimizations were performed at the BP86-D3(BJ)/def2-TZVP[S4-S12] level of theory, and electronic structure calculations at the B3LYP-D3(BJ)/def2-TZVPD[S4-S14] (or ωB97M-V/def2-TZVPD[S15,S16] if explicitly stated) level of theory. Vibrational frequencies were calculated with Turbomole-7.7 using *in vacuo* optimized structures on the same level of theory utilized in geometry optimization. They were converted into vibrational free energies at T = 298.15 K using the *thermo* submodule of the XTB software package[S17,S18] with an empirical scaling factor of 0.9914. Solvation free energies were computed using the COSMO-RS

method,[S19,S20] that combines the conductor-like solvation model (COSMO) and statistical fluid thermodynamics, using the COSMO*therm23* software package.[S21] Spin multiplicities of the ground electronic states were determined from the dependency of the spin-crossover energies on the exact exchange fraction, similar to the procedure outlined earlier.[S22] For this linear regression we used the BP86, PBE,[S11,S23] B3LYP*,[S24,S26] B3LYP and PBE0[S11,S23,S27] functionals, and the exact spin multiplicity of the ground state was determined at an exact exchange fraction of 15%, corresponding to the specialized B3LYP*[S28-S30] or PBE0*[S31,S32] functionals, which were found to reproduce high-quality CASPT2+δMRCI for similar metal complexes with high accuracy.[S31,S32] Following the reaction coordinate driven transition state search method,[S33,S34] one-dimensional PESs were calculated analogous to geometry optimizations. The exact position of a transition state pertaining to the electronic structure methods on a PES was determined from the Newton polynomial and confirmed by the presence of exactly one negative frequency.

Assessing the solvation free energy of a proton dissolved in acetonitrile under standard conditions is difficult with standard quantum chemical methods.[S35] Thus, we utilize the experimental estimate of $\Delta_S G(H^+) = -260.2$ kcal·mol^{-1} based on the cluster pair approximation (corresponding to the same standard states of 1 atm, T = 298.15 K and 1 mol·L^{-1}).[S36] As experimental reduction potentials are presented in reference to Fc^+/Fc, calculated reduction potentials are consistently displayed relative to a reference of 4.96 V.[S36] Finally, a correction of 1.9Δn kcal·mol^{-1} (corresponding to the difference between the concentration of the ideal gas at 298 K and 1 atm and its 1 mol·L^{-1} concentration; Δn is the change in the number of moles in the reaction)[S36-S38] has been applied so that the computed values refer to the 1 mol·L^{-1} standard state.

Microkinetic modelling was performed with the Tenua 2.1 software package.[S39] A period of 1 h reaction time was modelled, using a step length of 0.1 s. The numerical accuracy parameter ε was kept consistently at 10^{-5}. The initial concentration of CO_2 was chosen to be 0.28 M according to its concentration in saturated acetonitrile.[S40] Rate constants for the considered reaction steps were derived from transition state theory, using the Eyring equation[S41-S43] (eq. S2) extended by Wigner's tunneling correction[S44] (eq. S3), which utilizes the single complex vibrational frequency $\omega^{\ddagger}$ at the transition state. Within our model, catalyst reduction and the evolution of the protonated metal carboxylic acids were assumed to be instantaneous. We justify the former with its irrelevancy with respect to rate-controlling chemical reaction steps and the latter with the barrier-free nature of the process. Chemical binding of solvent and proton donor molecules to the reduced catalysts was

neglected due to low binding affinity. The formation of formate was neglected due to its low fraction in the experimental product spectrum under the employed experimental conditions (see main text). We refrained from calculating transition states for intermolecular PTs between the proton donor and various catalytic intermediates due to our inability to define a reasonable trajectory for the process without the use of extensive modelling of at least the first solvation shell, which is beyond the scope of our computational methodology. The free energy of transition states of intermolecular PTs was instead approximated via a Marcus relation[S45-S48] (eq. S4), using a range from 50 to 80 kcal·mol^{-1} for the intrinsic reaction barrier λ. For consistency, rate constants derived from this Marcus relation were multiplied by a factor of 2, which is in accordance with typical values for Wigner tunneling corrections determined for intramolecular PTs within this study. To analyze computed reaction rates of each elementary reaction step i, we used Campbell's degree of rate control[S49,S50] (eq. S5). For the numerical derivates involved, a change to forward (and backward) rate constants of each selected reaction step of 0.1 ppt was consistently applied to avoid numeric catastrophic cancellation, while providing a sufficiently small change to assume negligibility of higher order expansion terms (eq. S6). We sampled the degree of rate control as soon as the change in turnover frequency (TOF) of the respective product fell below 1 ppt to enforce maximal agreement with the framework of quasi-steady-state reaction kinetics.

$$k_i = \kappa_i \frac{k_B T}{h} e^{-\frac{\Delta G^\ddagger}{k_B T}} \tag{S2}$$

$$\kappa_i = \left(\frac{\hbar|\omega^\ddagger|}{2k_B T}\right) \frac{1}{\sin\left(\frac{\hbar|\omega^\ddagger|}{2k_B T}\right)} \tag{S3}$$

$$\Delta G^\ddagger = \frac{(\Delta G + \lambda)^2}{4\lambda} \tag{S4}$$

$$\chi_{C,i}(\text{Product}) = \frac{k_i}{\text{TOF(Product)}} \frac{\partial \text{TOF(Product)}}{\partial k_i}\bigg|_{K_i, k_{j\neq i}} \tag{S5}$$

$$\frac{df}{dk_i}\bigg|_{K_i, k_{j\neq i}} = \lim_{h\to 0} \frac{f(k_i+h, k_{-i}+h) - f(k_i)}{h} \approx \frac{f(1.0001\, k_i, 1.0001\, k_{-i}) - f(k_i, k_{-i})}{0.0001\, k_i} \tag{S6}$$

S2. Electrochemical section

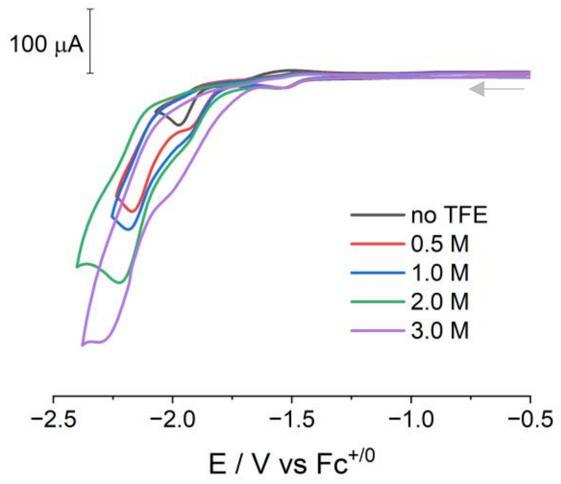

Figure S1. CVs of 1 mM complex **CoL** in acetonitrile under CO_2 in the presence of 0-3 M TFE measured at room temperature and at a scan rate of 0.1 V/s.

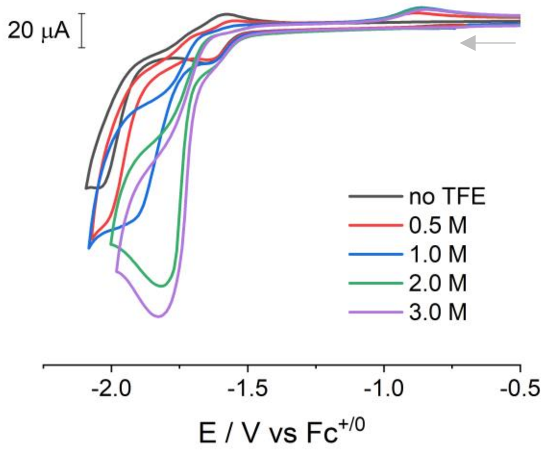

Figure S2. CVs of 1 mM complex **CoL^{OMe}** in acetonitrile under CO_2 in the presence of 0-3 M TFE measured at room temperature and at a scan rate of 0.1 V/s.

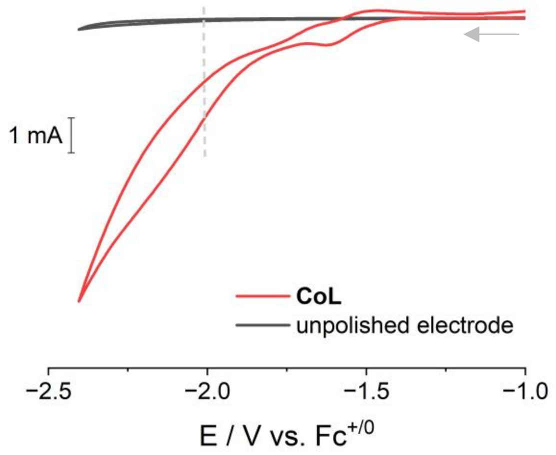

Figure S3. Comparison of the CVs obtained using the GC rod employed for the CPE experiments as the working electrode: 1 mM complex **CoL** in acetonitrile under CO_2 in the presence of 1 M TFE (red trace), unpolished electrode after 2 h CPE in acetonitrile under CO_2 in the presence of 1 M TFE (black trace). Scaling vs. $Fc^{+/0}$ was made by subtracting 0.4 V to the potential measured vs. SCE. The dashed grey line identified the applied potential in CPE.

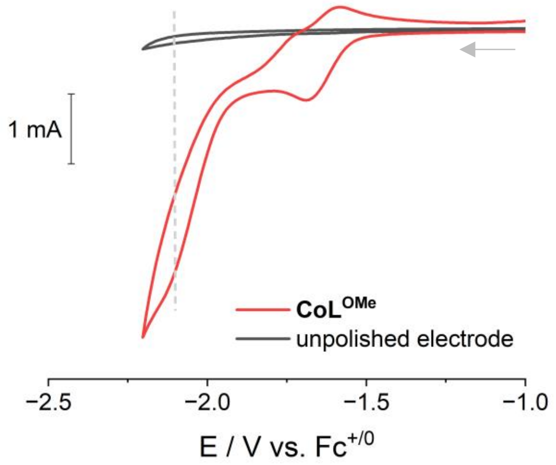

Figure S4. Comparison of the CVs obtained using the GC rod employed for the CPE experiments as the working electrode: 1 mM complex **CoL^{OMe}** in acetonitrile under CO_2 in the presence of 1 M TFE (red trace), unpolished electrode after 2 h CPE in acetonitrile under CO_2 in the presence of 1 M TFE (black trace). Scaling vs. $Fc^{+/0}$ was made by subtracting 0.4 V to the potential measured vs. SCE. The dashed grey line identified the applied potential in CPE.

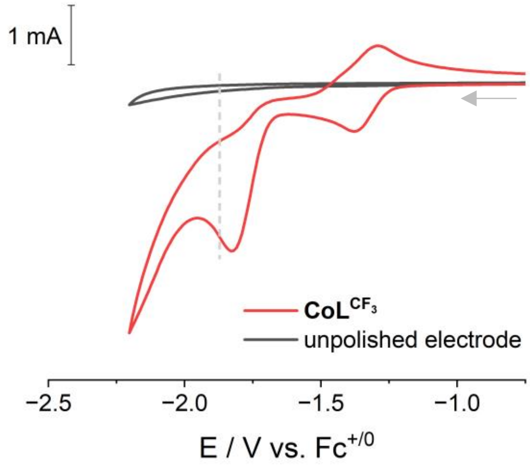

Figure S5. Comparison of the CVs obtained using the GC rod employed for the CPE experiments as the working electrode: 1 mM complex **CoL^{CF_3}** in acetonitrile under CO_2 in the presence of 1 M TFE (red trace), unpolished electrode after 2 h CPE in acetonitrile under CO_2 in the presence of 1 M TFE (black trace). Scaling vs. $Fc^{+/0}$ was made by subtracting 0.4 V to the potential measured vs. SCE. The dashed grey line identified the applied potential in CPE.

Table S1. Relevant electrochemical data.

	$E_{red,1}$ / V [a]	$E_{red,2}$ / V [a]	E_{cat} / V [b]	i_{cat}/i_p
**CoL**	−1.50	−2.06	−1.91	4.9
**CoL^{CF_3}**	−1.29	−1.73, −1.85	−1.73, −1.85	1.7, 2.5
**CoL^{OMe}**	−1.59	−2.13	−1.98	5.1

[a] Measured by CV in acetonitrile under Ar and referenced vs. Fc^+/Fc; [b] measured under CO_2 as the potential at the inflection point of the catalytic wave and referenced vs. Fc^+/Fc.

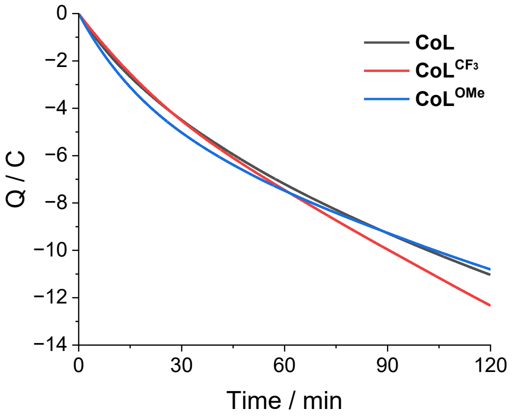

a)

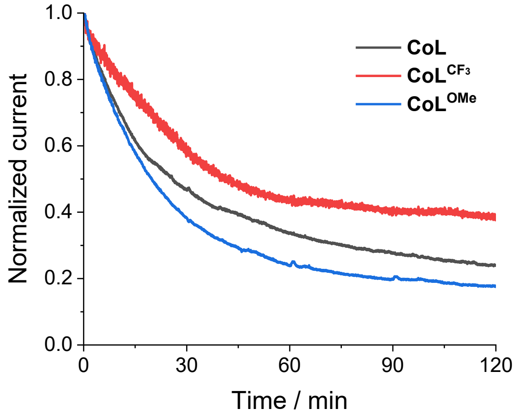

b)

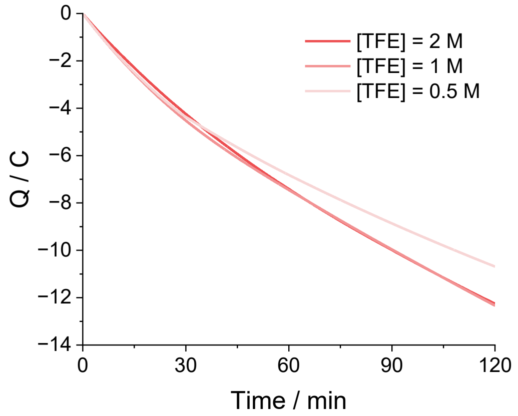

c)

Figure S6. a) Coulometric traces obtained through CPE of 1 mM complex in acetonitrile under CO_2 in the presence of 1 M TFE and b) the corresponding normalized chronoamperometric profiles; c) coulometric traces obtained through CPE of 1 mM **CoL^{CF_3}** in acetonitrile under CO_2 in the presence of 0.5-2 M TFE.

S3. Computational section

Table S2. Calculated reduction potentials and deviation from experimental values in parentheses.

	**$E_{red,1}$ / V vs Fc^+/Fc**	**$E_{red,2}$ / V vs Fc^+/Fc**
**CoL**	–1.53 (–0.03)	–2.07 (–0.01)
**CoL^{CF_3}**	–1.20 (+0.09)	–1.69 (+0.04)
**CoL^{OMe}**	–1.65 (–0.06)	–2.11 (–0.02)

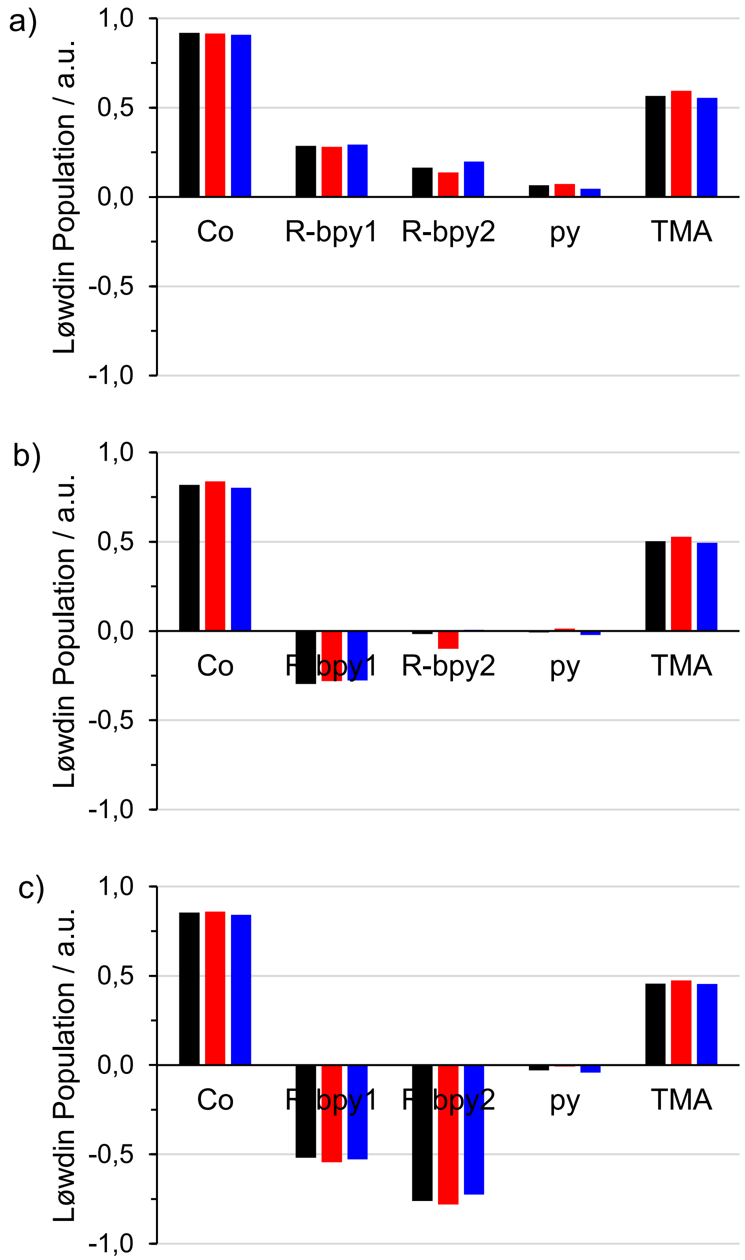

Figure S7. Løwdin population analysis summed over all atoms of respective chemical moieties (R-bpy1 denotes the bipyridine moiety facing the coordination site, and R-bpy2 the one facing away from it, TMA stands for trimethylenamine) in **CoL** (black), **CoL$^{\mathbf{CF_3}}$** (red), and **CoL$^{\mathbf{OMe}}$** (blue) for their: (a) unreduced (charge +2) quartet ground state; (b) one-electron reduced (charge +1) triplet ground state; (c) two-electron reduced (uncharged) quartet ground state.

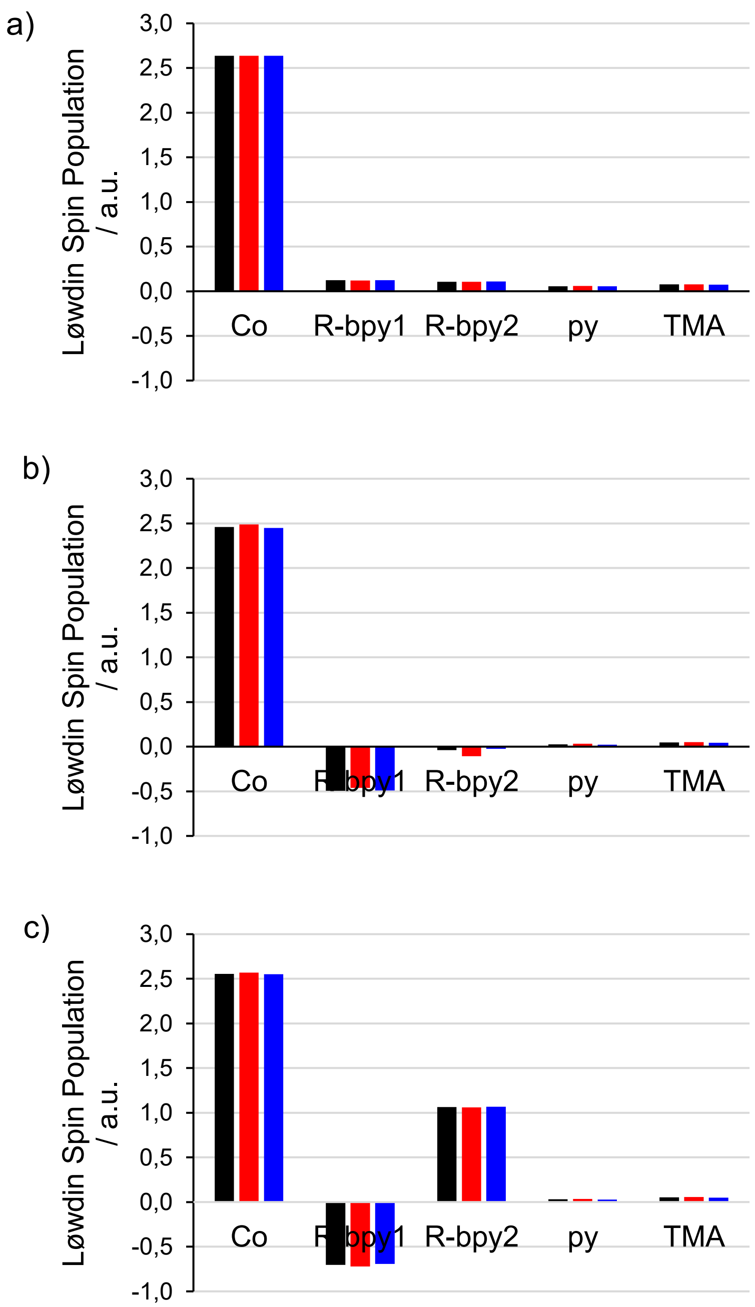

Figure S8. Løwdin *spin* population analysis summed over all atoms of respective chemical moieties (R-bpy1 denotes the bipyridine moiety facing the coordination site, and R-bpy2 the one facing away from it, TMA stands for trimethylenamine) in **CoL** (black), **CoL**$^{\mathbf{CF_3}}$ (red), and **CoL**$^{\mathbf{OMe}}$ (blue) for their: (a) unreduced (charge +2) quartet ground state; (b) one-electron reduced (charge +1) triplet ground state; (c) two-electron reduced (uncharged) quartet ground state.

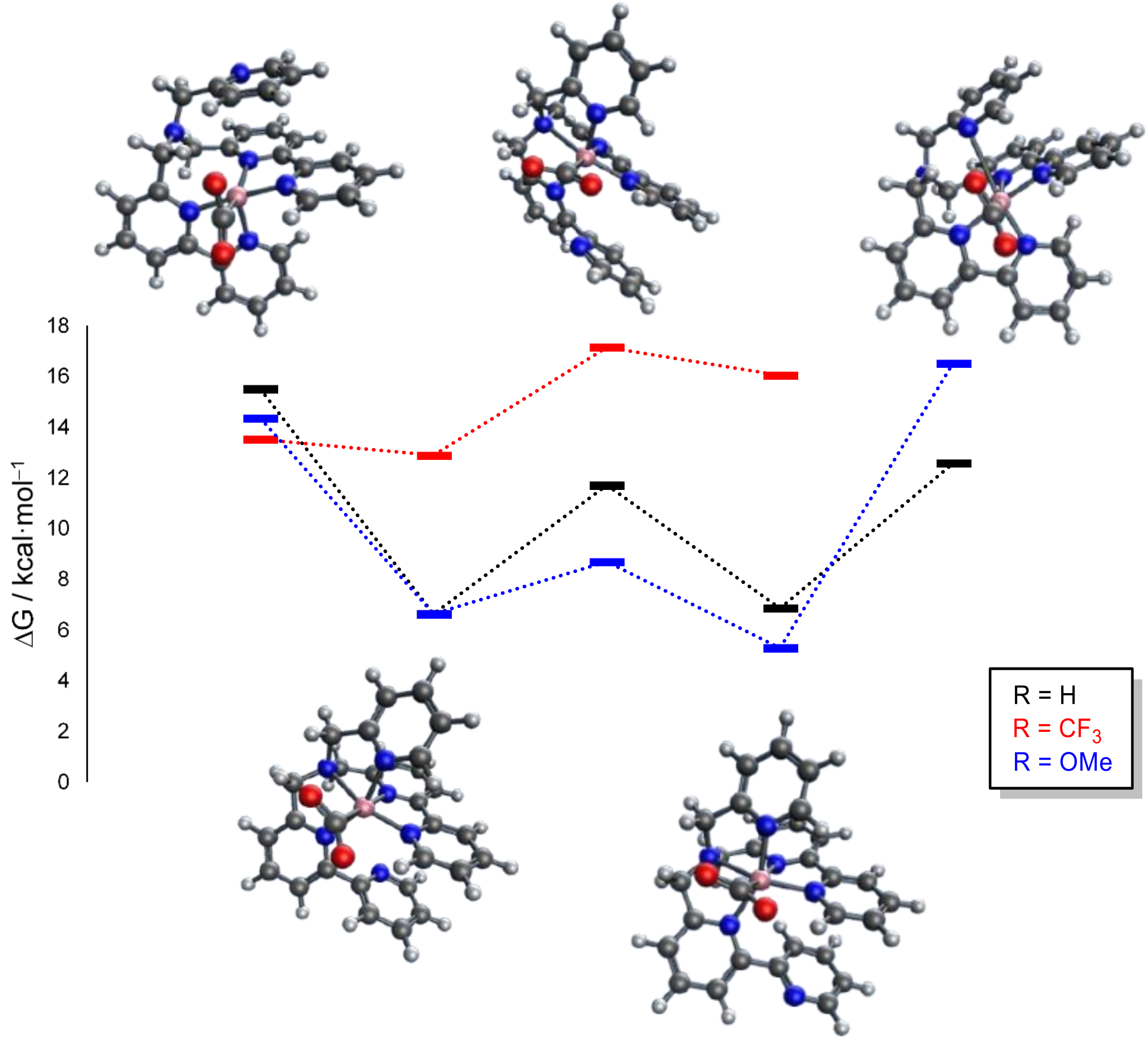

Figure S9. Binding free energy / kcal·mol^{-1} of CO_2 with **CoL** (black), **CoL^{CF_3}** (red), and **CoL^{OMe}** (blue) in the heptacoordinated form (far right) and in different conformers with a detached pyridine moiety. The heptacoordinated form of **CoL^{CF_3}** could not be evaluated under the employed computational protocol due to spontaneous substrate detachment in geometry optimizations.

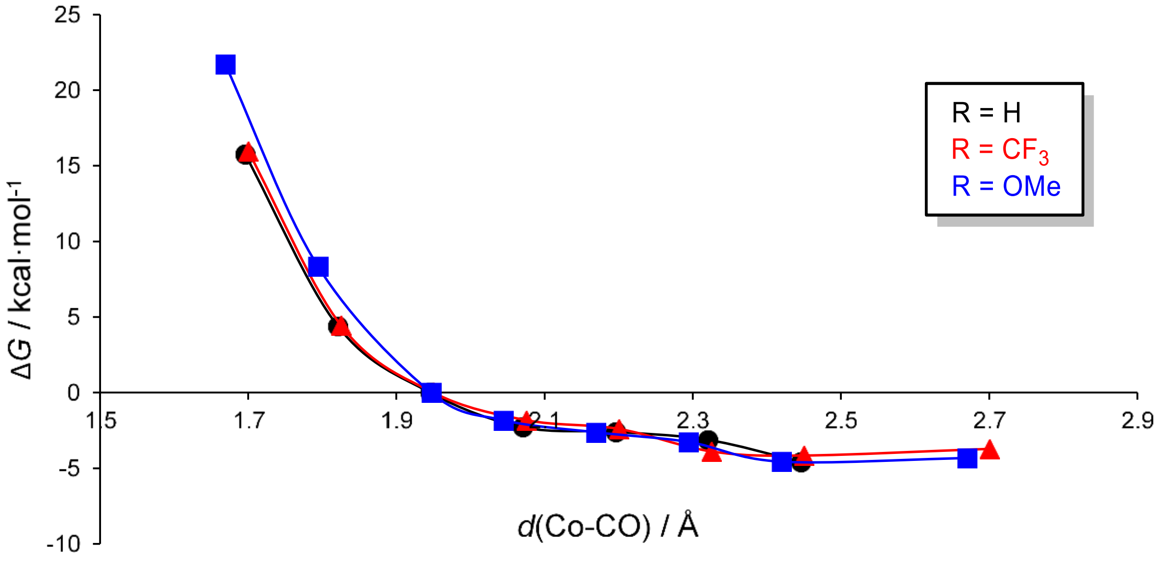

Figure S10. Potential free energy surface of the distance between CO and the central Co atom in **CoL** (black), **CoL**^{CF_3} (red), and **CoL**^{OMe} (blue). All structures show barrier-free detachment of CO at the B3LYP-D3/COSMO-RS (and ωB97M-V/COSMO-RS) level of theory, but feature a minor barrier at the BP86-D3/COSMO-RS level of theory, used in the geometry optimization. The minimum structure of the BP86-D3/COSMO-RS surface was used as reference geometry for this free energy surface. For free energies of CO removal from the respective catalysts in reference to this geometry see Scheme 2 of the main article.

Table S3. Calculated pK_a values of the metal hydrides formed by protonation of the metal centers of **CoL**, **CoL^{CF_3}**, and **CoL^{OMe}**, and their respective free energies of formation according to eq. S1. Further displayed are free energies of transition states of the respective metal hydride formations calculated using eq. S4 with different intrinsic reaction barriers.

**CoL^R**	**R = H**	**R = CF$_3$**	**R = OMe**
**pK_a**	13.6	4.7	15.8
**ΔG / kcal·mol^{-1}**	24.7	36.9	21.7
**$\Delta G^‡(\lambda=50)$ / kcal·mol^{-1}**	27.9	37.7	25.7
**$\Delta G^‡(\lambda=60)$ / kcal·mol^{-1}**	29.9	39.1	27.8
**$\Delta G^‡(\lambda=70)$ / kcal·mol^{-1}**	32.0	40.8	30.0
**$\Delta G^‡(\lambda=80)$ / kcal·mol^{-1}**	34.2	42.7	32.3

Scheme S1. Alternative reaction mechanism of the CO_2RR for our cobalt catalysts involving pyridine protonation, calculated at the B3LYP-D3(BJ)/COSMO-RS/def2-TZVPD level of theory. Reaction free energies (ΔG), in kcal·mol^{-1}, of individual reaction steps are printed along the reaction arrows for R = H (black), for R = CF_3 (red), and for R = OMe (blue). Free energies of associated transition states ($\Delta G^{\ddagger}$), in kcal·mol^{-1}, are added in parentheses. Free energies of intermolecular proton transfers assume the deprotonation of TFE.

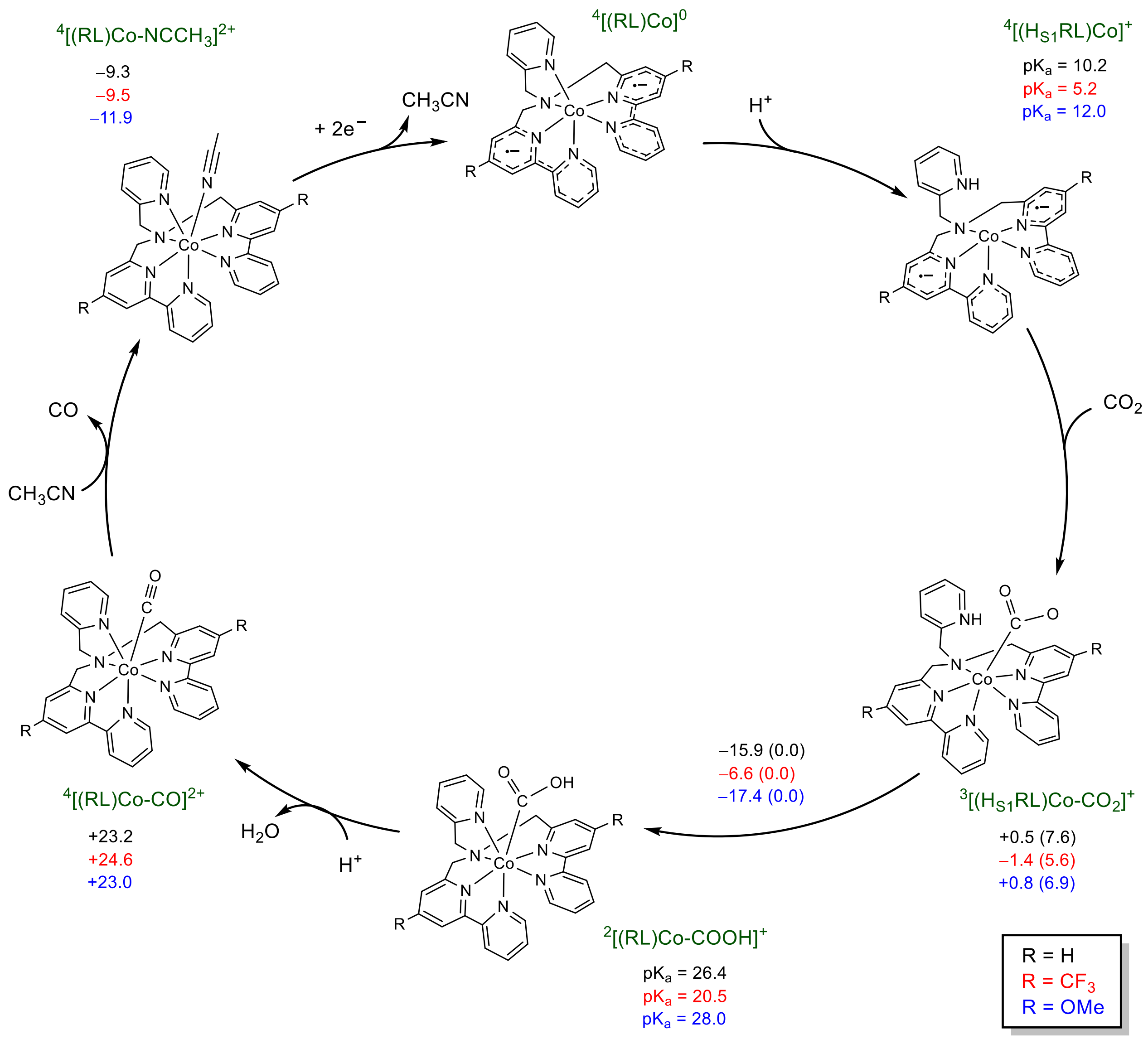

Scheme S2. Alternative reaction mechanism of the HER for our cobalt catalysts involving pyridine protonation, calculated at the B3LYP-D3(BJ)/COSMO-RS/def2-TZVPD level of theory. Reaction free energies (ΔG), in kcal·mol^{-1}, of individual reaction steps are printed along the reaction arrows for R = H (black), for R = CF_3 (red), and for R = OMe (blue). Free energies of associated transition states ($\Delta G^{\ddagger}$), in kcal·mol^{-1}, are added in parentheses. Free energies of intermolecular proton transfers assume the deprotonation of TFE.

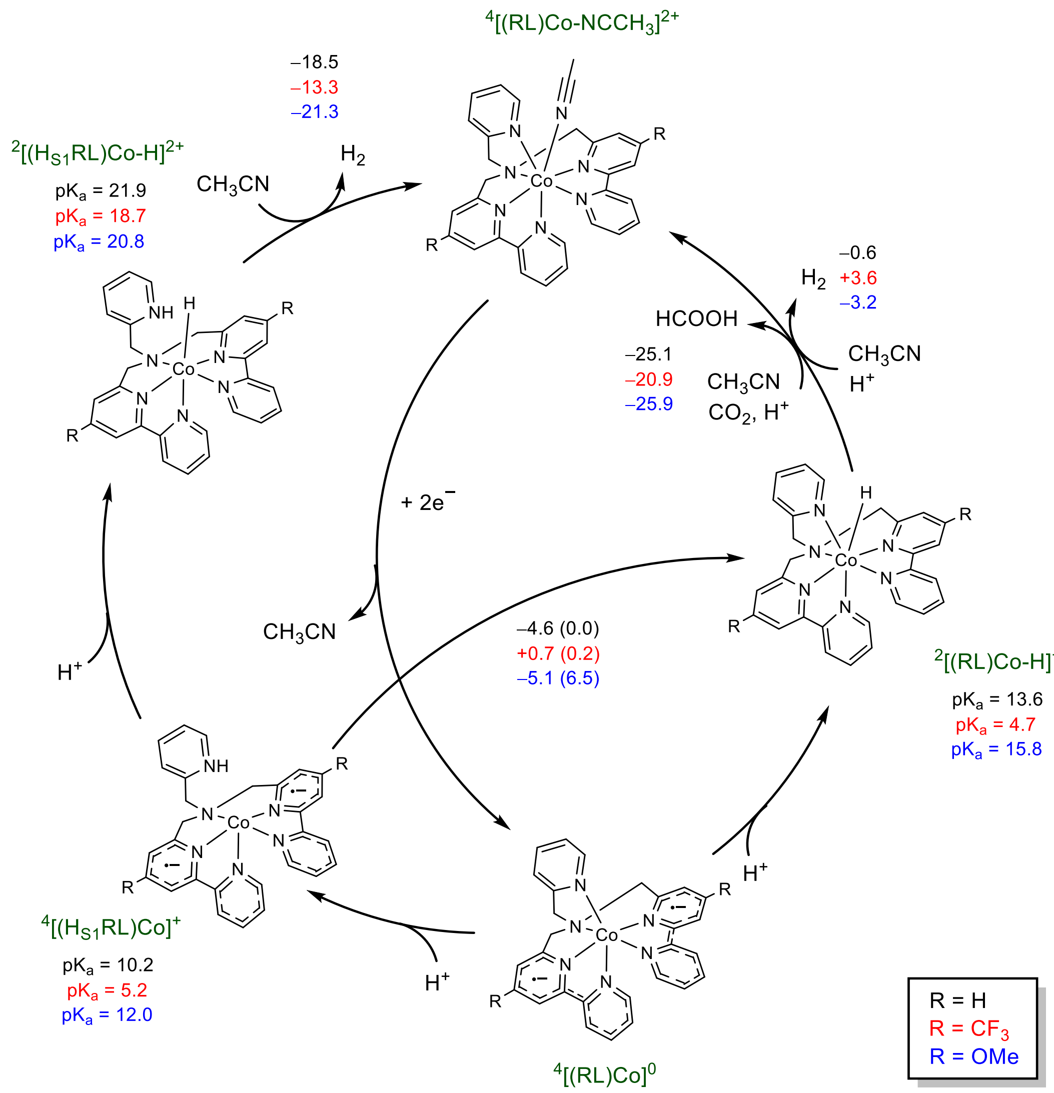

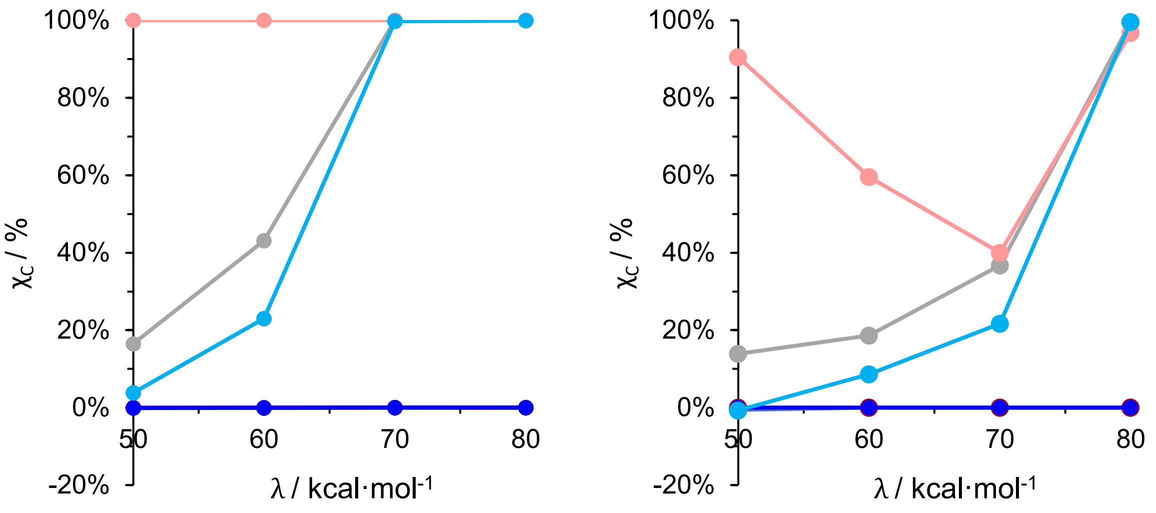

Figure S11. Degree of rate control distribution w.r.t. the formation of CO (dark) and H_2 (light) calculated as a function of the intrinsic reaction barrier λ of the Marcus relation (eq. S4) for the protonation of the metal center of **CoL** (black), **CoL^{CF_3}** (red), and **CoL^{OMe}** (blue): results from microkinetic modelling without the inclusion of ligand protonation (left), results including ligand protonation (right).

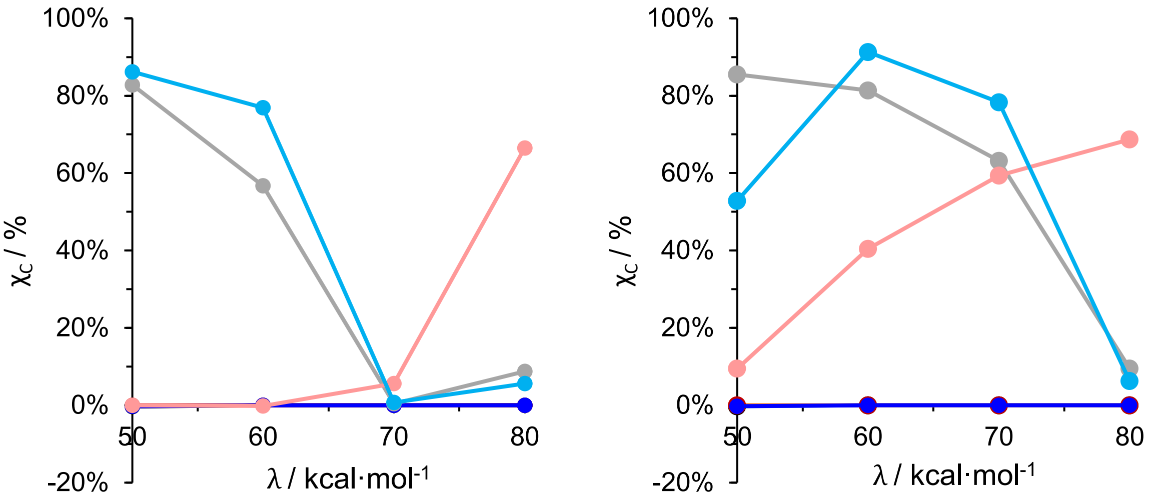

Figure S12. Degree of rate control distribution w.r.t. the formation of CO (dark) and H_2 (light) calculated as a function of the intrinsic reaction barrier λ of the Marcus relation (eq. S4) for the protonation of the metal hydride of **CoL** (black), **CoL^{CF_3}** (red), and **CoL^{OMe}** (blue): results from microkinetic modelling without the inclusion of ligand protonation (left), results including ligand protonation (right).

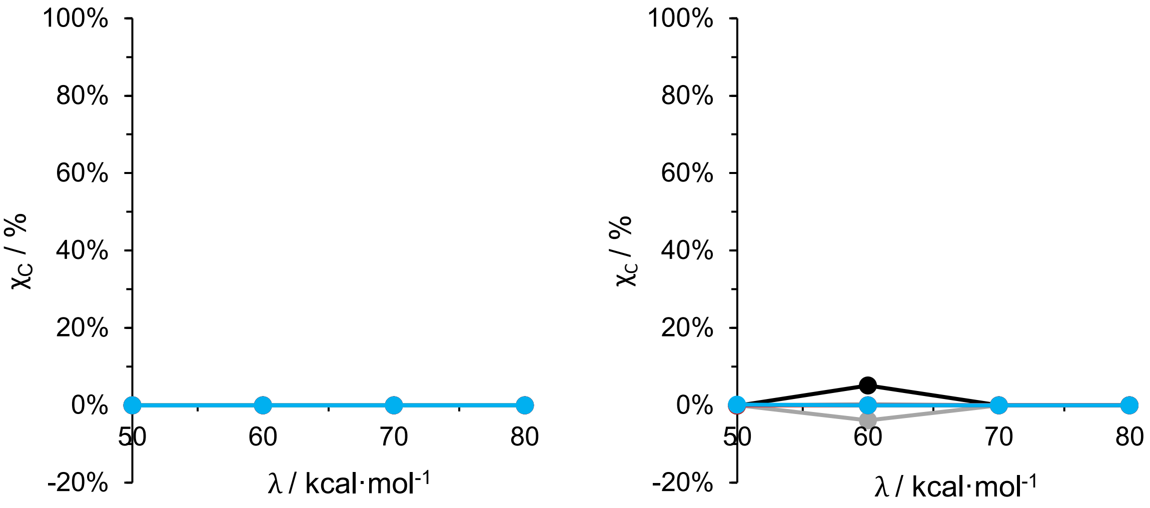

Figure S13. Degree of rate control distribution w.r.t. the formation of CO (dark) and H_2 (light) calculated as a function of the intrinsic reaction barrier λ of the Marcus relation (eq. S4) for the binding of CO_2 to **CoL** (black), **CoL^{CF_3}** (red), and **CoL^{OMe}** (blue): results from microkinetic modelling without the inclusion of ligand protonation (left), results including ligand protonation (right).

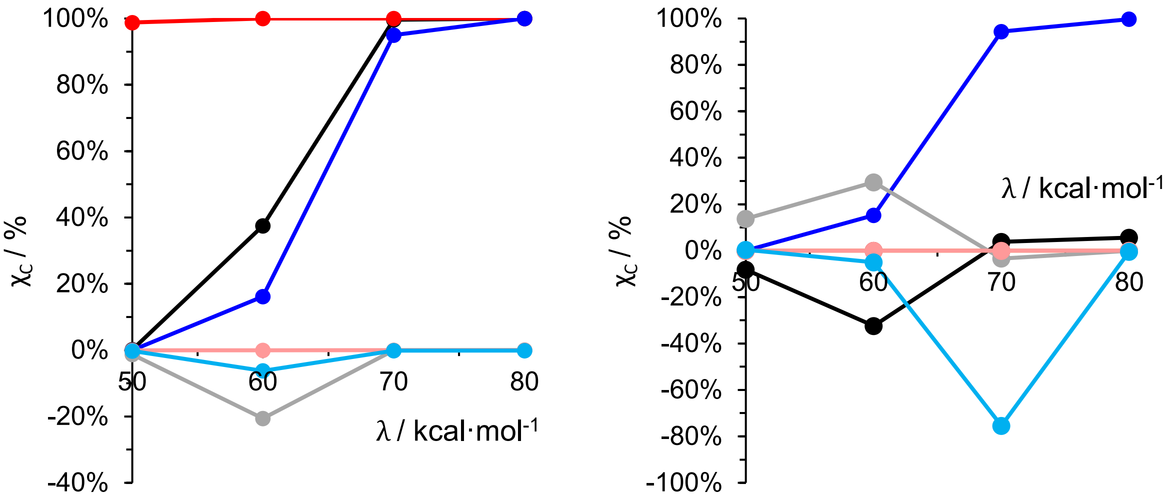

Figure S14. Degree of rate control distribution w.r.t. the formation of CO (dark) and H_2 (light) calculated as a function of the intrinsic reaction barrier λ of the Marcus relation (eq. S4) for the protonation of the intermediate metal carboxylate of **CoL** (black), **CoL^{CF_3}** (red), and **CoL^{OMe}** (blue): results from microkinetic modelling without the inclusion of ligand protonation (left), results including ligand protonation (right).

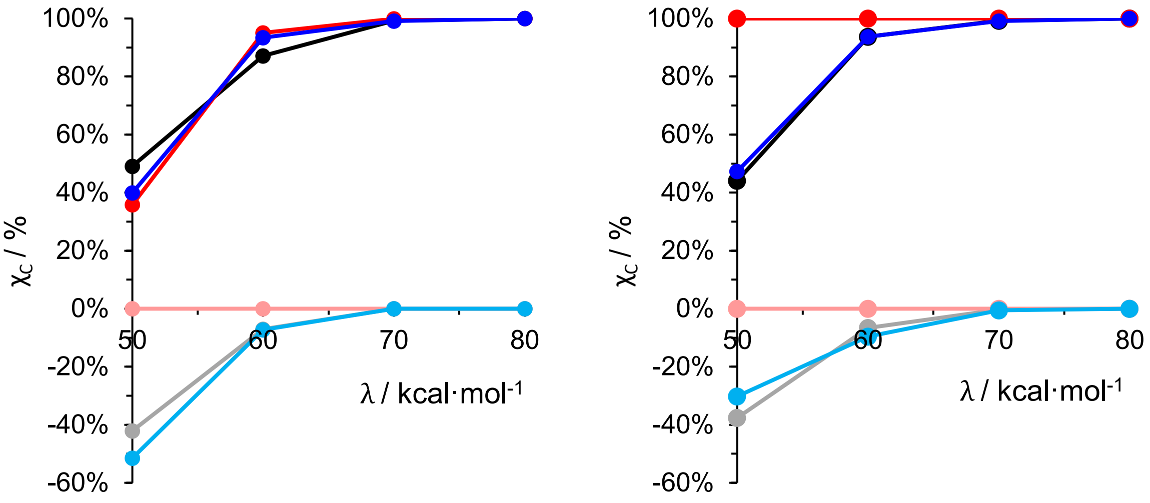

Figure S15. Degree of rate control distribution w.r.t. the formation of CO (dark) and H_2 (light) calculated as a function of the intrinsic reaction barrier λ of the Marcus relation (eq. S4) for the protonation of the metal carboxylic acid intermediate of **CoL** (black), **CoL^{CF_3}** (red), and **CoL^{OMe}** (blue): results from microkinetic modelling without the inclusion of ligand protonation (left), results including ligand protonation (right).

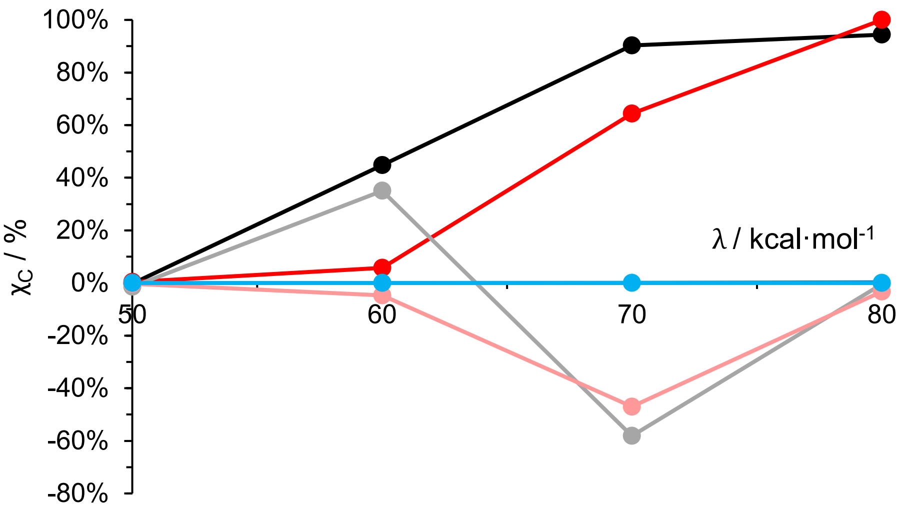

Figure S16. Degree of rate control distribution w.r.t. the formation of CO (dark) and H_2 (light) calculated as a function of the intrinsic reaction barrier λ of the Marcus relation (eq. S4) for the protonation of the DBPy-PyA ligand of **CoL** (black), **CoL**$^{\mathbf{CF_3}}$ (red), and **CoL**$^{\mathbf{OMe}}$ (blue). Values for the protonation of the **A1** and **A2** conformers have been combined.

S4. Photochemical section

The photochemical activity was evaluated by considering diverse key performance indicators that can be extrapolated from the kinetics traces of CO_2RR.

1) The total amount of the products (CO, H_2 and formate) estimated at the plateau of the kinetics.
2) The maximum turnover number (TON) is estimated by eq. S7, where the n_{max} and n_{cat} are the maximum moles of product and the moles of the catalyst, respectively.

$$TON = \frac{n_{max}}{n_{cat}} \tag{S7}$$

3) The initial rate of products (CO and H_2) production (r, mol·s^{-1}) calculated from the slope in the linear portion of the kinetic trace.
4) The turnover frequencies (TOF, s^{-1}) are defined according to eq. S8.

$$TOF = \frac{r}{n_{cat}} = \frac{TON}{t} \tag{S8}$$

The quantum yield (Φ) calculated according to eq. S9 where Φ is the quantum yield, according to eq S3 where φ is the absorbed photon flux (Einstein·s^{-1}).

$$\Phi = \frac{r}{\varphi} \tag{S9}$$

The quantification of the absorbed photon flux (φ) was obtained by employing the photoreaction between $[Ru(bpy)_3]Cl_2 \cdot 6H_2O$ and 9,10-diphenylanthracene (DPA) in aerated acetonitrile solution as an actinometer, following an established protocol.[S51] A 6 mL solution consisting of 0.4 mM $[Ru(bpy)_3]Cl_2 \cdot 6H_2O$ and 0.1 mM DPA in acetonitrile was prepared and its UV-Vis spectrum was recorded. Subsequently, the solution was subjected to a one-minute irradiation using the same light source and setup employed in the photochemical experiments. A new UV-Vis spectrum of the irradiated sample was then acquired. Both samples were diluted for the UV-Vis spectra measurements by mixing 1 mL of the sample with 2 mL of acetonitrile. The moles of DPA

consumed were computed following eq. S10 where A_i is the absorbance at 372 nm before the irradiation, A_f is the absorbance at 372 nm after the irradiation, V is the volume of the sample (considering dilution) and ε_{372nm} = 11,100 $M^{-1}cm^{-1}$ is the attenuation coefficient of DPA at 372 nm in acetonitrile. A cuvette with a 1 cm path length was employed.

$$n\ DPA\ consumed = \frac{A_i - A_f}{\varepsilon_{372\ nm}} \times V \tag{S10}$$

The absorbed photon flux (φ) was calculated using eq. S11 where Φ_{ref} = 0.019 is the quantum yield of the actinometer and t = 60 s is the irradiation time.

$$\varphi = \frac{n\ DPA\ consumed}{\Phi_{ref}\ t} \tag{S11}$$

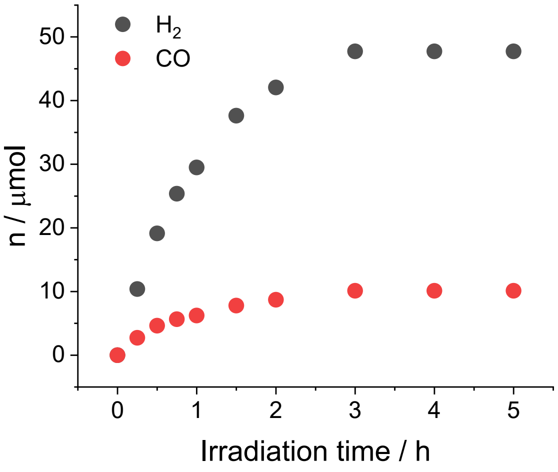

a)

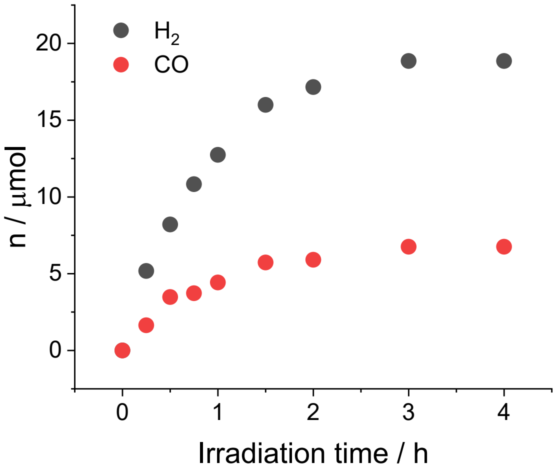

b)

Figure S17. Kinetics of product formation upon irradiation (460-nm LED) of an acetonitrile solution containing 0.4 mM [Ru(bpy)$_3$](PF$_6$)$_2$, 0.1 M DIPEA, and 1 M TFE under CO_2 in the presence of a) 50 µM and b) 10 µM **CoL**.

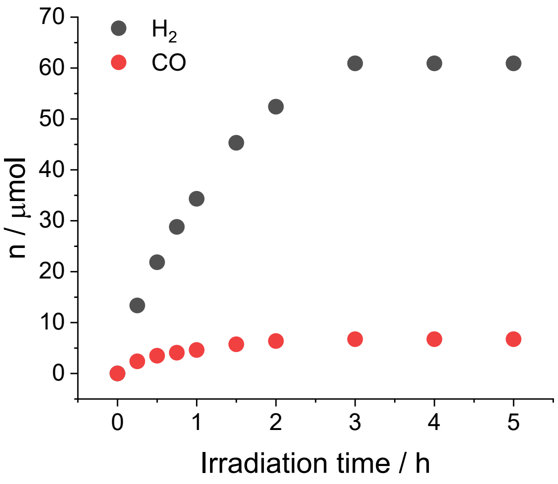

a)

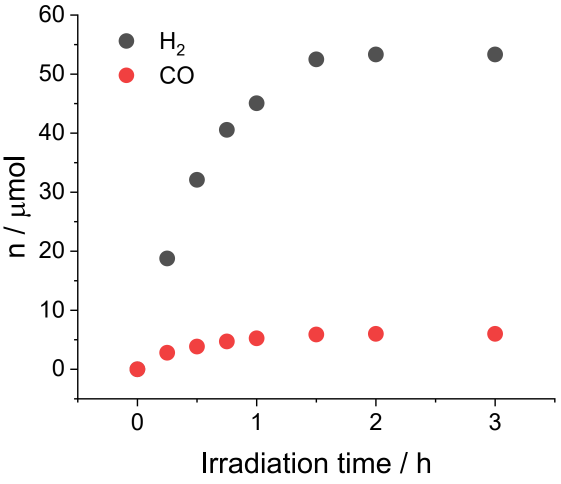

b)

Figure S18. Kinetics of product formation upon irradiation (460-nm LED) of an acetonitrile solution containing 0.4 mM [Ru(bpy)$_3$](PF$_6$)$_2$, 0.1 M DIPEA, and 1 M TFE under CO_2 in the presence of a) 50 µM and b) 10 µM **CoL^{OMe}**.

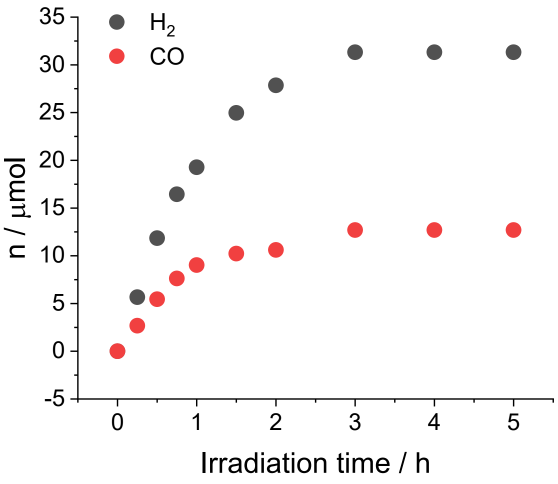

a)

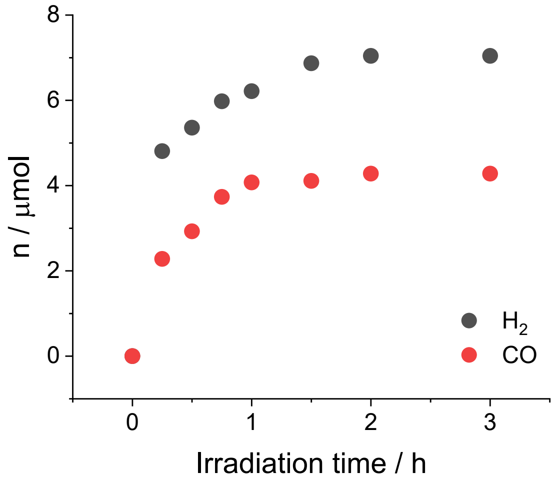

b)

Figure S19. Kinetics of product formation upon irradiation (460-nm LED) of an acetonitrile solution containing 0.4 mM [Ru(bpy)$_3$](PF$_6$)$_2$, 0.1 M DIPEA, and 1 M TFE under CO_2 in the presence of a) 50 µM and b) 10 µM **CoL^{CF_3}**.

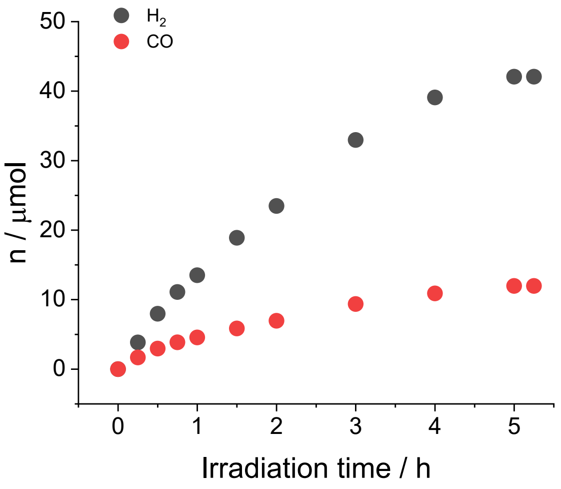

Figure S20. Kinetics of product formation upon irradiation (460-nm LED) of an acetonitrile solution containing 50 µM **CoL**, 0.4 mM 4DPAIPN, 0.1 M DIPEA, and 1 M TFE under CO_2.

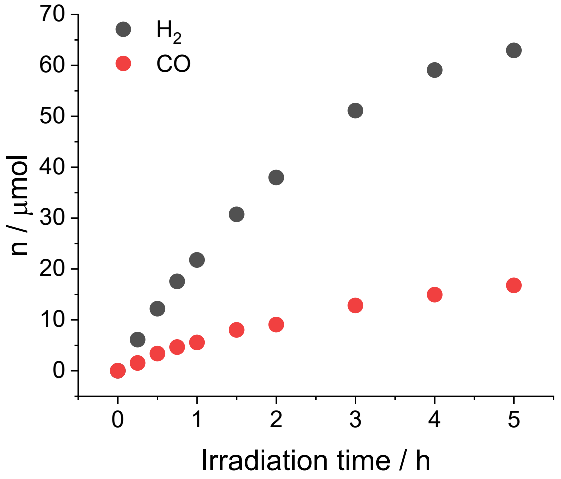

Figure S21. Kinetics of product formation upon irradiation (460-nm LED) of an acetonitrile solution containing 50 µM **CoL^{OMe}**, 0.4 mM 4DPAIPN, 0.1 M DIPEA, and 1 M TFE under CO_2.

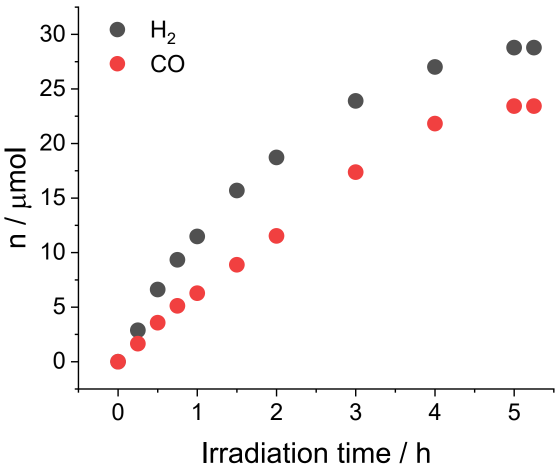

Figure S22. Kinetics of product formation upon irradiation (460-nm LED) of an acetonitrile solution containing 50 µM **CoL**^{CF_3}, 0.4 mM 4DPAIPN, 0.1 M DIPEA, and 1 M TFE under CO_2.

Table S4. Control experiments.[a]

		n / µmol [j]		
**Entry**	**PS**	**H_2**	**CO**	**Formate**
1 [b]	$[Ru(bpy)_3]^{2+}$	-	-	-
2 [b]	4DPAIPN	-	-	-
3 [c]	$[Ru(bpy)_3]^{2+}$	-	-	-
4 [c]	4DPAIPN	-	-	-
5 [d]	$[Ru(bpy)_3]^{2+}$	-	-	-
6 [d]	4DPAIPN	-	-	-
7 [e]	$[Ru(bpy)_3]^{2+}$	0.8	2.2	1.4
8 [e]	4DPAIPN	2.1	0.3	-
9 [f, g]	$[Ru(bpy)_3]^{2+}$	3.9	-	-
10 [f, g]	4DPAIPN	79.7	-	-
11 [f, h]	$[Ru(bpy)_3]^{2+}$	6.5	-	-
12 [f, h]	4DPAIPN	72.8	-	-
13 [f, i]	$[Ru(bpy)_3]^{2+}$	5.8	-	-
14 [f, i]	4DPAIPN	53.6	-	-

[a] 460-nm LED, 0.1 M DIPEA, 0.4 mM PS, 1 M TFE, 50 µM catalyst in acetonitrile after 20 min bubbling of CO_2; [b] no light; [c] no DIPEA; [d] no PS; [e] no catalyst; [f] degassed using Ar instead of CO_2; [g] **CoL** used as a catalyst; [h] **CoL^{CF_3}** used as a catalyst; [i] **CoL^{OMe}** used as a catalyst; [j] estimated after 5 h of irradiation.

S5. References of the Supporting Information

S1 Connelly, N. G.; Geiger, W. E. *Chem. Rev.* **1996**, *96*, 877-910.

S2 Droghetti, F.; Villa, L.; Sartorel, A.; Dell'Amico, L.; Ruggi, A.; Natali, M. *ChemSusChem* **2025**, *18*, e202402627.

S3 Balasubramani, S. G., et al. *J. Chem. Phys.* **2020**, *152*, 184107.

S4 Grimme, S.; Antony, J.; Ehrlich, S.; Krieg, H. *J. Chem. Phys.* **2010**, *132*, 154104.

S5 Grimme, S.; Ehrlich, S.; Goerigk, L. *J. Comp. Chem.* **2011**, *32*, 1456-1465.

S6 Becke, A. D.; Johnson, E. R. *J. Chem. Phys.* **2005**, *122*, 154101.

S7 Johnson, E. R.; Becke, A. D. *J. Chem. Phys.* **2005**, *123*, 024101.

S8 Johnson, E. R.; Becke, A. D. *J. Chem. Phys.*, **2006**, *124*, 174104.

S9 Becke, A. D. *Phys. Rev. A* **1988**, *38*, 3098-3100.

S10 Perdew, J. P. *Phys. Rev. B* **1986**, *33*, 8822-8824.

S11 Slater, J. C. *Phys. Rev.* **1951**, *81*, 385-390.

S12 Vosko, S. H.; Wilk, L.; Nusair, M. *Can. J. Phys.* **1980**, *58*, 1200-1211.

S13 Lee, C.; Yang, W.; Parr, R. G. *Phys. Rev. B* **1988**, *37*, 785-789.

S14 Stephens, P. J.; Devlin, F. J.; Chabalowski, C. F.; Frisch, M. J. *J. Phys. Chem.* **1994**, *98*, 11623-11627.

S15 Mardirossian, N.; Head-Gordon, M. *J. Chem. Phys.* **2016**, *144*, 214110.

S16 Vydrova, O. L.; Van Voorhis, T. *J. Chem. Phys.* **2010**, *133*, 244103.

S17 Bannwarth, C.; Caldeweyher, E.; Ehlert, S.; Hansen, A.; Pracht, P.; Seibert, J.; Spicher, S.; Grimme, S. *WIREs Comput. Mol. Sci.*, **2020**, *11*, e1493.

S18 Grimme, S. *Chem. Eur. J.* **2012**, *18*, 9955-9964.

S19 Klamt, A.; Jonas, V.; Burger, T.; Lohrenz, J. C. W. *J. Phys. Chem. A* **1998**, *102*, 5074-5085.

S20 Eckert, F.; Klamt, A. *AIChE J.* **2002**, *48*, 369-385.

S21 Eckert, F.; Klamt, A. COSMO*therm*, Version C2.1, Release 01.10; COSMO*l*ogic GmbH & Co KG, Leverkusen, Germany, **2009**.

S22 Bowman, D. N.; Jakubikova, E. *Inorg. Chem.* **2012**, *51*, 6011-6019.

S23 Perdew, J. P.; Burke, K.; Ernzerhof, M. *Phys. Rev. Lett.* **1996**, *77*, 3865-3868.

S24 Reiher, M.; Salomon, O.; Artur Hess, B. *Theor. Chem. Acc.* **2001**, *107*, 48-55.

S25 Salomon, O.; Reiher, M.; Hess, B. A. *J. Chem. Phys.* **2002**, *117*, 4729-4737.

S26 Becke, A. D. *J. Chem. Phys*. **1993**, *98*, 5648-5652.

S27 Adamo, C.; Barone, V. *J. Chem. Phys.* **1999**, *110*, 6158-6170.

S28 Reiher, M.; Salomon, O.; Artur Hess, B. *Theor. Chem. Acc*. **2001**, *107*, 48-55.

S29 Reiher, M. *Inorg. Chem*. **2002**, *41*, 6928-6935.

S30 Salomon, O.; Reiher, M.; Hess, B. A. *J. Chem. Phys.* **2002**, *117*, 4729-4737.

S31 Reimann, M.; Kaupp, M. *J. Chem. Theory Comput.* **2022**, *18*, 7442-7456.

S32 Reimann, M.; Kaupp, M. *J. Chem. Theory Comput.* **2023**, *19*, 97-108.

S33 Scharfenberg, P. *J. Comput. Chem*. **1982**, *3*, 277-282.

S34 Berente, I.; Náray-Szabó, G. *J. Phys. Chem. A* **2006**, *110*, 772-778.

S35 Kastenholz, M. A.; Hünenberger, H. *J. Chem. Phys*. **2006**, *124*, 224501.

S36 Kelly, C. P.; Cramer, C. J.; Truhlar, D. G. *J. Phys. Chem. B* **2007**, *2*, 408-422.

S37 Khobragade, D. A.; Mahamulkar, S. G.; Pospíšil, L.; Císařovμ, I.; Rulíšek, L.; Jahn, U. *Chem. Eur. J*. **2012**, *18*, 12267-12277.

S38 Ben-Naim, A. Solvation Thermodynamics; Plenum: New York, **1987**.

S39 Tenua 2.1 the kinetics simulator for Java, Copyright 2025, Daniel Wachsstock, MD

S40 Tomita, Y.; Teruya, S.; Koga, O.; Hori, Y. *J. Electrochem. Soc.* **2000**, *147*, 4164-4167.

S41 Glass, S.; Laidler, K.; Eyring, H. The Theory of Rate Processes, McGraw-Hill, New York, **1941**.

S42 Laidler, K.; Tweedale, A. *Adv. Chem. Phys*. **1971**, *21*, 113.

S43 Hofacker, L. *Int. J. Quantum Chem*. **1969**, *35*, 33.
Garrett, B. C.; Truhlar, D. G. *J. Phys. Chem*. **1979**, *83*, 1052-1079.

S44 E. P. Wigner, *Z. Phys. Chem*. **1932**, *19*, 203.

S45 Bolton, J. R.; Archer, M. D. *Adv. Chem. Ser*. **1991**, *228*, 7-23.

S46 Marcus, R. A.; Sutin, N. *Biochim. Biophys. Acta, Rev. Bioenerg*. **1985**, *811*, 265-322.

S47 Liu, Z. Y.; Wei, Z. C.; Chou, P. T. *J. Phys. Chem. A* **2021**, *125*, 6611-6620.

S48 Mayer, J. M. *J. Phys. Chem. Lett*. **2011**, *2*, 1481-1489.

S49 Stegelmann, C.; Andreasen, A.; Campbell, C. T. *J. Am. Chem. Soc*. **2009**, *131*, 8077-8082.

S50 Campbell, C. T. *J. Catal.* **2001**, *204*, 520-524.

S51 Pitre, S. P.; McTiernan, C. D.; Vine, W.; DiPucchio, R.; Grenier, M.; Scaiano, J. C. *Sci. Rep.* **2015**, *5*, 16397.
